# Stromal netrin 1 coordinates renal arteriogenesis and mural cell differentiation

**DOI:** 10.1242/dev.201884

**Published:** 2023-11-24

**Authors:** Peter M. Luo, Xiaowu Gu, Christopher Chaney, Thomas Carroll, Ondine Cleaver

**Affiliations:** ^1^ Department of Molecular Biology, University of Texas Southwestern Medical Center, 5323 Harry Hines Blvd., Dallas, TX 75390, USA; ^2^ Center for Regenerative Science and Medicine, University of Texas Southwestern Medical Center, 5323 Harry Hines Blvd., Dallas, TX 75390, USA; ^3^ Internal Medicine and Division of Nephrology, University of Texas Southwestern Medical Center, 5323 Harry Hines Blvd., Dallas, TX 75390, USA

**Keywords:** Netrin 1, Klf4, Artery, Mural cell, SMA, NG2, Pericyte, Nephron, Foxd1, Mouse

## Abstract

The kidney vasculature has a complex architecture that is essential for renal function. The molecular mechanisms that direct development of kidney blood vessels are poorly characterized. We identified a regionally restricted, stroma-derived signaling molecule, netrin 1 (Ntn1), as a regulator of renal vascular patterning in mice. Stromal progenitor (SP)-specific ablation of Ntn1 (*Ntn1^SPKO^*) resulted in smaller kidneys with fewer glomeruli, as well as profound defects of the renal artery and transient blood flow disruption. Notably, Ntn1 ablation resulted in loss of arterial vascular smooth muscle cell (vSMC) coverage and in ectopic SMC deposition at the kidney surface. This was accompanied by dramatic reduction of arterial tree branching that perdured postnatally. Transcriptomic analysis of *Ntn1^SPKO^* kidneys revealed dysregulation of vSMC differentiation, including downregulation of Klf4, which we find expressed in a subset of SPs. Stromal Klf4 deletion similarly resulted in decreased smooth muscle coverage and arterial branching without, however, the disruption of renal artery patterning and perfusion seen in *Ntn1^SPKO^*. These data suggest a stromal Ntn1-Klf4 axis that regulates stromal differentiation and reinforces stromal-derived smooth muscle as a key regulator of renal blood vessel formation.

## Teaser

**Summary:** Netrin 1 secreted by cortical stromal progenitors in the kidney regulates vascular patterning of the renal arterial tree, as well as vascular smooth muscle cell differentiation via Klf4.

## INTRODUCTION

The human kidney filters over 200 l of blood daily, removing wastes and excess fluid via urine production. This process requires a complicated vascular network, including smaller capillaries of the glomerulus and surrounding nephrons that participate in solute exchange, as well as a large tree-like arterial network that channels blood throughout the organ (Mohamed and Sequiera-Lopez, 2019). We previously characterized the semi-stereotyped branching of the initial renal arterial tree, which becomes distinguishable as a tributary of the abdominal aorta at embryonic day (E) 13 in mouse (Daniel et al., 2018). The question arises as to how renal vessels take on the proper architecture during development. It is likely that the various non-vascular cells present during nephrogenesis lay down a patchwork of cues that direct vascular patterning; however, these interactions remain unknown.

The stroma, or interstitium, of the kidney plays a crucial role in kidney development and patterning. Derived primarily from a population of Foxd1^+^ progenitors present at the kidney periphery, stromal cells permeate the developing kidney, encasing both epithelial and endothelial structures. Far more than simply providing a supportive matrix for kidney to grow within, the stroma participates in cellular crosstalk required for cell fate and overall tissue morphogenesis (Das et al., 2013; Hurtado et al., 2015; Ide et al., 2018; Mendelsohn et al., 1999; Zhang et al., 2003). Stromal progenitors (SPs) communicate with nephron progenitors (NPCs) to regulate their collective differentiation and with the ureteric bud (Ub) to direct collecting duct formation and branching (Paroly et al., 2013).

The stroma is also required for development of the renal vasculature (Hum et al., 2014), but it is unclear how. SPs may signal directly to vascular precursors, and/or they may regulate endothelial cells via their descendants within the perivascular niche, collectively termed ‘mural cells’ (Sequeira-Lopez et al., 2015). Mural cells are heterogeneous along the vasculature. They include vascular smooth muscle cells (vSMCs) that densely enwrap larger caliber vessels and pericytes that invest more sparsely along capillaries (Armulik et al., 2010; Jain, 2003). Mural cells physically support endothelial cells as they differentiate and experience increasing shear stress and pressure from hemodynamic flow (Gaengel et al., 2009); they regulate a host of vascular properties, including vessel patency, quiescence and tone (Stratman et al., 2017); and they have recently been proposed to regulate the patterning and development of vasculature (Orlich et al., 2022).

Development of organ blood vessels is guided by various angiogenic factors, including the secreted laminin-like protein netrin 1 (Ntn1). Ntn1 has been shown to regulate blood vessel development and endothelial cell migration, function and survival via signaling to Unc5b (Boyé et al., 2022; Larrivée et al., 2007; Castets et al., 2009; Lu et al., 2004). Ntn1 also signals to non-endothelial cells, however, as it is also required for axonal migration, lung and pancreatic branching, and other key processes in organ development (Bin et al., 2015; Brunet et al., 2014; Colamarino and Tessier-Lavigne, 1995; Liu et al., 2004; Matilainen et al., 2007; Mediero et al., 2015; Yebra et al., 2003; Yung et al., 2015). Ntn1 mediates these effects via signaling to multiple other receptors, including other members of the Unc5 family, Neo1 and DCC (Freitas et al., 2008; Huyghe et al., 2020).

Here, we examine the unknown role of Ntn1 in kidney development, focusing on the renal vasculature. We identified Ntn1 expression in the kidney stroma using single cell RNA-sequencing (scRNA-seq) (England et al., 2020) and confirmed this observation *in vivo*. In addition, we show renal expression of numerous Ntn1 receptors, positioning Ntn1 as a potential mediator of stromal crosstalk to other cell types, including the vasculature. Using conditional genetic ablation, we show that Ntn1 is required for renal vascular patterning and for normal distribution of vSMCs. Mechanistically, we show that loss of Ntn1 results in loss of the transcription factor Klf4, which is a key regulator of smooth muscle progenitor fate. Loss of either Ntn1 or Klf4 results in ectopic mural cells in cortical stroma, disruption of arterial smooth muscle coverage and decreased arterial branching. Together, these findings underscore a previously unreported role for Ntn1 in kidney development, specifically for patterning and mural cell coverage of the renal vasculature.

## RESULTS

### Cortical Foxd1^+^ stromal progenitors secrete Ntn1 during development

To investigate the role of Ntn1 in the developing kidney, we assessed both its transcript and protein expression throughout nephrogenesis. *Ntn1* expression was observed by *in situ* hybridization within SPs located in the renal cortex, from E12.5 to E14.5 (Fig. S1A-C′). Transcripts were not detected in the medullary region (Med) or in the nephrogenic zone, which includes Ub tips and NPCs (Fig. S1C′). Publicly available *in situ* data for *Foxd1* (Fig. S1D) shows the location of SPs in the developing kidney, showing that *Ntn1* is expressed in a subset of these progenitors. Ntn1 receptors Unc5c (Fig. S1E) and Unc5b (Fig. S1F) were expressed in NPCs and blood vessels, respectively, and Neo1 (Fig. S1G) was expressed in multiple lineages, including epithelial cells and cortical stroma.

**Fig. 1.**
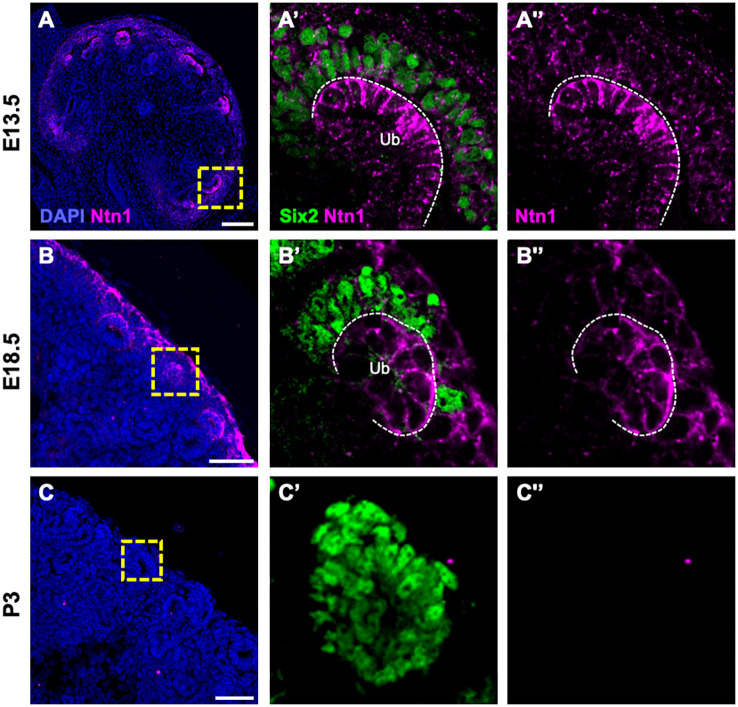
**Ntn1 protein is expressed by cortical stromal progenitors during embryonic kidney development.** (A-C) Immunofluorescence for Ntn1 on E13.5, E18.5 and P3 kidney sections. (A′-C′) Magnification of boxed areas showing co-stains for Six2 to delineate nephron progenitor cells, located under stromal progenitors and surrounding the ureteric bud tip (Ub, outlined). (A″-C″) Single channel images. Scale bars: 100 µm (A); 50 µm (B,C).

Ntn1 protein, on the other hand, was present within the stroma and amongst Six2^+^ NPCs throughout the cortex of the developing kidney (Fig. 1A-B″; Fig. S1H-K,N,N′). Ntn1 protein also appeared to be enriched at Ub tips; however, no transcripts were detected in Ub tips via *in situ* hybridization or scRNA-seq (Fig. S1N′). These data suggest that Ntn1 is secreted by SPs into kidney cortex. Over the course of development, Ntn1 remained restricted to the nephrogenic zone up to E18.5 (Fig. S1H-K). Protein levels decreased rapidly after birth and were no longer detectable by postnatal day (P) 3 (Fig. 1C-C″; Fig. S1L,M), a timepoint corresponding to the cessation of nephrogenesis. Together, these results support a role for stromal Ntn1 during kidney development.

### Loss of Ntn1 impairs kidney development

**Fig. 2.**
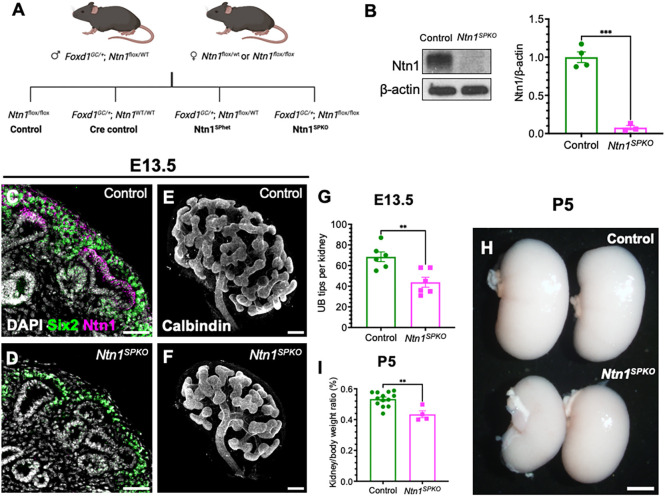
**Ablation of Ntn1 in the kidney stroma impairs kidney development.** (A) Breeding scheme of stromal progenitor-specific knockout of Ntn1 (*Ntn1^SPKO^*) using *Foxd1^GC^*. (B) Western blot and quantification for Ntn1 in *Ntn1^SPKO^* kidney lysates (*n*=3, ***P=0.0001). (C,D) Immunofluorescence for Ntn1 and Six2 on E13.5 kidney sections. (E,F) Whole-mount immunofluorescence of E13.5 kidney ureteric tree with calbindin. (G) Quantification of ureteric bud tip counts (*n*=6, **P=0.0044). (H) Photo of P5 control and mutant kidneys. (I) Kidney weight/body weight comparison for P5 pups (*n*=4, **P=0.0016). Each *n*=1 embryo, multiple litters represented per experiment. Bar graphs show mean±s.e.m. *P*-values calculated by unpaired two-tailed *t*-test. Scale bars: 50 µm (C-F); 1 mm (H).

Global deletion of Ntn1 results in embryonic lethality (Bin et al., 2015; Yung et al., 2015). To test its function in the developing kidney, we generated a kidney stroma-specific deletion of Ntn1 (hereafter termed *Ntn1^SPKO^*) by crossing either *Ntn1^f/f^* or *Ntn1^f/+^* females to *Ntn1^f/+^* heterozygote males containing a *Foxd1^GC^* allele (Fig. 2A) (Kobayashi et al., 2014). We confirmed Ntn1 deletion by both western blot analysis of whole embryonic kidneys (Fig. 2B) and immunofluorescence (IF) (Fig. 2C,D). Importantly, no Ntn1 protein was detectable in *Ntn1^SPKO^* kidneys, indicating that all protein found in the cortex was derived from SPs.

Grossly, *Ntn1^SPKO^* kidneys were smaller than those of littermate control embryos (Fig. 2E,F). Analysis of kidney and embryo lengths at E13.5 showed that decrease in kidney size was independent of potential overall embryonic growth delays (Fig. S2A). We analyzed Ub branching in E13.5 *Ntn1^SPKO^* kidneys using whole-mount IF (WMIF) for calbindin and observed significantly fewer epithelial tips (Fig. 2E-G). Notably, Six2^+^ NPCs remained present in the nephrogenic zone without Ntn1, although the number appeared to be mildly reduced (Fig. 2C,D). Staining for *Dolichos biflorus* agglutinin (DBA) and Pax8 at P5 showed relatively normal morphology of ureteric and nephrogenic epithelia, respectively (Fig. S2B,C). However, Six2^+^ NPCs were observed in *Ntn1^SPKO^* kidneys as late as P5, whereas control kidneys experienced cessation of nephrogenesis and NPC depletion (Fig. S2D,E,F). Perduring Six2^+^ cells in *Ntn1^SPKO^* kidneys expressed NCAM, a cell adhesion molecule that indicates progression towards the renal vesicle stage of nephrogenesis (Fig. S2D,E). These data suggest that without Ntn1 a proportion of NPCs are maintained past the timepoint when they are normally depleted, but are still capable of undergoing mesenchymal-to-epithelial transition (MET).

Mutant kidneys continued to be smaller after birth, both by visual comparison at P5 (Fig. 2H; Fig. S2G) and by kidney/body weight ratios (Fig. 2I; Fig. S2G). To assess the effect of Ntn1 loss on nephron generation, we performed acid maceration of adult kidneys followed by glomerular counting and found significantly fewer glomeruli in *Ntn1^SPKO^* kidneys (Fig. S2H). Glomerular counts correlated with kidney/body weight ratio, suggesting that this decrease was associated with the decrease in kidney size (Fig. S2I). These data show that prolonged NPC presence does not result in an increase in the number of nephrogenesis cycles.

### Impaired arteriogenesis and vascular patterning occurs without Ntn1

**Fig. 3.**
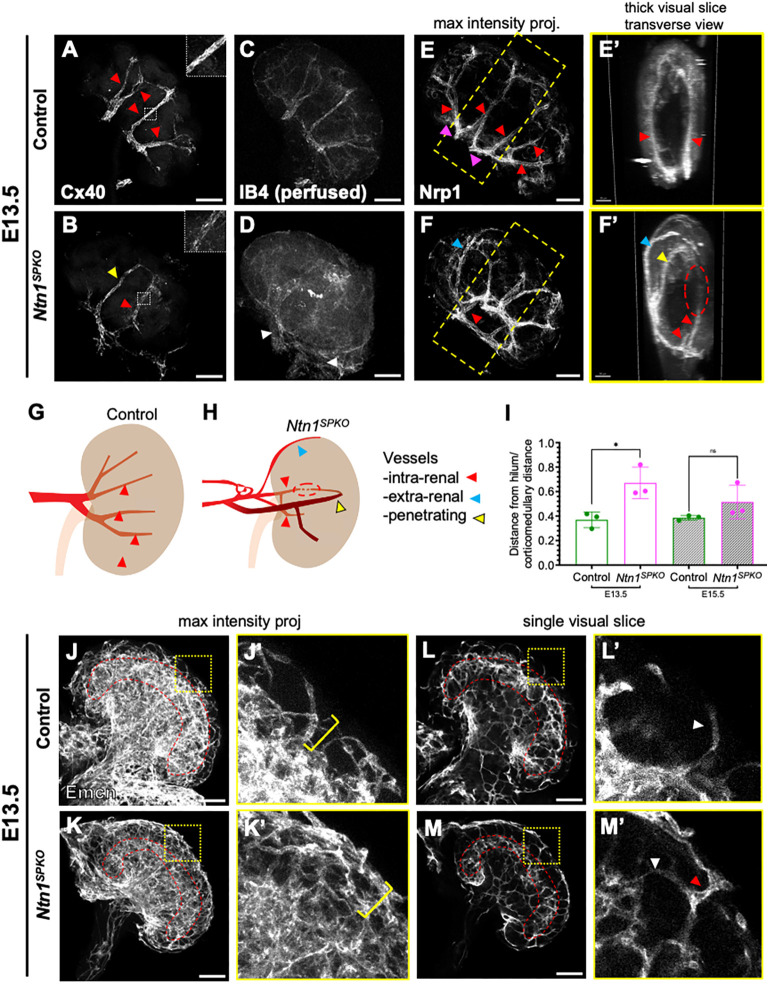
***Ntn1^SPKO^* exhibits altered patterning of early renal vasculature, resulting in decreased blood flow into the kidney.** (A,B) Whole-mount immunofluorescence (WMIF) of E13.5 kidneys for arteries remodeling under flow using Cx40. Yellow arrowhead, artery entering the kidney from the cortex (‘penetrating’); red arrowheads, arteries entering from the hilum (‘intra-renal’, see inset). (C,D) WMIF of perfused isolectin B4 (IB4), showing perfusion of arteries. White arrowheads, IB4 signal in arteries outside of mutant kidney. (E-F′) WMIF of arterial marker Nrp1 in E13.5 kidneys showing total arterial tree morphology. Yellow and red arrowheads, penetrating and intra-renal arteries, respectively; blue arrowheads, artery travelling along ventral kidney surface (‘extra-renal’). Yellow box represents width of 200 µm slice in E′-F′, viewed orthogonally, through center of Nrp1 WMIF. Dashed red circle in mutant indicates likely connection between penetrating and intra-renal arteries. (G,H) Model of arterial patterning defects in control and *Ntn1*^*SPKO*^ kidneys, showing intra renal, extra-renal and penetrating arteries. (I) Comparison of renal artery invasion of the hilum, measured by the average distance from the point of entry to the center of the ureter at the hilum divided by the total corticomedullary distance of the kidney, at E13.5 (*n*=3, *P=0.0221) and E15.5 (*n*=3, *P*=0.1823). (J-K′) WMIF for Emcn marking capillary vasculature in E13.5 kidneys. Cortical vascular plexus under the nephrogenic zone is outlined in red. Magnification of boxed areas (J′,K′) show capillary architecture surrounding the nephrogenic zone (yellow brackets). (L-M′) Single slice images of Emcn WMIF showing cortical vascular plexus (red outlines) and vessels along the kidney surface in *Ntn1^SPKO^*. Magnification of boxed areas (L′,M′) show contribution and caliber of capillaries in the nephrogenic zone. Red arrowhead, large capillary deriving from the surface in mutant; white arrowheads, small capillaries deriving from vascular plexus. Each *n*=1 embryo, multiple litters represented per experiment. Bar graphs show mean±s.e.m. *P*-values calculated by unpaired two-tailed *t*-test. Scale bars: 100 µm (A-F,J-M); 50 µm (E′-F′).

Ntn1 is known to regulate angiogenesis and vascular patterning in other organs (Larrivee et al., 2007; Park et al., 2004; Wilson et al., 2006). We asked whether Ntn1 regulates development of renal blood vessels. WMIF of E13.5 kidneys for mature arteries with connexin 40 (Cx40; also known as Gja5) showed reduced expression within *Ntn1^SPKO^* kidney vessels (Fig. 3A,B). As Cx40 is expressed in arteries in response to blood flow (Chong et al., 2011), we injected fluorescently tagged isolectin B4 (IB4) into the embryonic vasculature at E13.5 to assess vessel perfusion. In control kidneys, IB4 remained within renal arteries, but in *Ntn1^SPKO^* kidneys, no specific signal was evident within most of the kidney; instead we noted IB4 perfusion of large vessels outside the kidney (Fig. 3C,D).

We performed WMIF with a pan-arterial marker neuropilin (Nrp1) (You et al., 2005) to assess arterial architecture. Arteries were present, but grossly mispatterned in mutant kidneys, especially with respect to renal artery invasion into the kidney (Fig. 3E-F′). Control renal arteries consist of two main arms (magenta arrowheads) that enter the kidney at the hilum and form a ramifying tree-like structure while remaining within the kidney (Fig. 3E,E′, red arrowheads). By contrast, *Ntn1^SPKO^* arteries often traveled along the surface (‘extra-renal arteries’, blue arrowheads), sometimes entering the kidney directly through the cortex to the middle of the kidney (termed ‘penetrating arteries’, yellow arrowheads), bypassing the hilum (Fig. 3F,F′).

Arteries in *Ntn1^SPKO^* kidneys that did enter via the hilum (‘intrarenal’ arteries) did not progress normally into the kidney (Fig. 3F,F′, red arrowheads). Maximum intensity projections of the center third of Nrp1 WMIFs showed significantly shorter intrarenal arterial length and excess extrarenal arteries in *Ntn1^SPKO^* kidneys (Fig. S3A-D). In some cases, penetrating arteries looped and connected with intra-renal arteries (Fig. 3E′,F′, red circle). Penetrating arteries displayed stronger Cx40 staining than intrarenal arteries in *Ntn1^SPKO^* kidneys, suggesting that flow may be shunted away from the hilum (Fig. 3B, yellow arrowhead). In addition, we observed collateral arteries between intrarenal arteries near the hilum of *Ntn1^SPKO^* kidneys (Fig. S3B, orange arrowheads), connecting the main renal artery branches improperly and potentially limiting flow into the kidney. In control kidneys, branches connect distally at the cortex via arcuate arteries (Fig. S3A, white arrowheads).

To further assess arterial fate, we performed WMIF for Sox17 at E13.5. Arteries in *Ntn1^SPKO^* kidneys expressed lower, but detectable Sox17, indicating that arterial identity was not completely disrupted without Ntn1 (Fig. S3E,F).

To assess the origin of penetrating vessels and the causes of arterial mispatterning, we performed immunostaining on whole abdominal arterial systems to define the path of the renal artery from the aorta. In control embryos, the renal artery forms a single large branch from the aorta that bifurcates at the hilum (Fig. S3G, gray arrowhead). In *Ntn1^SPKO^* embryos, we observed an ‘arterial plexus’ (red arrowhead) between the aorta and the kidney, resulting in early misdirection of arteries and in penetrating arteries (Fig. S3H, yellow arrowhead). We also observed multiple aortic branches contributing to the renal vasculature via ectopic collaterals (Fig. S3H, white arrowheads). Together, these results suggest a failure of vessels to remodel into a single renal artery, resulting in a network of misdirected vessels that likely leads to decreased perfusion. These defects in proximal arterial patterning and their effects on renal arteries are summarized in a graphical model (Fig. 3G,H).

Next, we analyzed the progression of defects in proximal artery patterning and invasion. Intriguingly, arteries in control and *Ntn1^SPKO^* kidneys exhibited similar levels of Cx40 immunostaining and IB4 perfusion by E15.5 (Fig. S3I-L). We performed WMIF for alpha smooth muscle actin (αSMA) to assess arterial invasion, using αSMA^+^ ureteric smooth muscle to mark the hilum (Fig. S3M,N). In *Ntn1^SPKO^* kidneys, penetrating arteries were present but entered the kidney closer to the hilum compared with controls at E13.5, sometimes making large turns to direct blood towards the cortex (Fig. S3N, yellow arrowhead). We measured the average distance from each arterial entry point to the ureter and divided it by the total corticomedullary distance. At E13.5, this ratio was significantly increased in mutants, but by 15.5 it became insignificant, suggesting that growth of the kidney progressively minimizes the effects of early arterial mispatterning, explaining the restoration of Cx40 and IB4 positivity (Fig. 3I).

By E18.5, effects of early arterial mispatterning on intrarenal vessels in *Ntn1^SPKO^* kidneys largely resolve. WMIF for αSMA showed a relatively normal branched tree, however a few abnormal arteries (white arrowheads) remain at the hilum of the kidney (Fig. S3O-P″).

### Ntn1 regulates both early renal arterial patterning and later ramification

Together, these results suggest that stromal Ntn1 is required for proper remodeling of the proximal renal artery between the aorta and the kidney during early nephrogenesis. Without Ntn1, improper arterial patterning and invasion results in a transient decrease in blood flow into the kidney. These defects are minimized as the kidney grows, restoring blood flow into the arterial tree. However, we note that the arterial tree is still defective, even at late stages. *Ntn1^SPKO^* kidneys displayed thinner arteries (Fig. S3O,P, inset) at E18.5. In addition, we counted branches of main renal arteries, excluding bifurcations and arteries leading directly to glomeruli, at E15.5 using αSMA WMIF. We observed fewer branches in *Ntn1^SPKO^* kidneys (Fig. S3Q), and figure 5 in Honeycutt et al. (2023), a parallel study, shows that arterial tree branching is significantly disrupted through adulthood. As decreases in tree-like branching continue well after blood flow is restored into the kidney, we suspect that they may not be caused by early mispatterning and transient loss of perfusion, but rather a separate process downstream of Ntn1.

### Loss of Ntn1 results in aberrant nephrogenic zone capillary vasculature

In addition to the arterial tree, the early kidney is populated by a dense vascular plexus that likely arises from endothelial progenitors within the metanephric mesenchyme (Daniel et al., 2018). At E13.5, these fine capillaries form a cortical plexus beneath the nephrogenic zone, which itself is relatively avascular to promote NPC differentiation (Daniel et al., 2018; Munro et al., 2017).

We analyzed capillary vascular plexus organization in *Ntn1^SPKO^* kidneys with WMIF for endomucin (Emcn) (Fig. 3J-M′). Whereas in control kidneys, the nephrogenic zone is relatively devoid of vessels (Fig. 3J,J′, yellow bracket), the cortex of *Ntn1^SPKO^* kidneys is densely packed with capillaries (Fig. 3K,K′, yellow bracket). Single visual slices of WMIFs show that vessels sprouting from the cortical vascular plexus in control kidneys are small in caliber (Fig. 3L,L′, white arrowhead), whereas in *Ntn1^SPKO^* larger capillaries populate the surface and connect to the cortical vascular plexus (Fig. 3M,M′, white and red arrowheads). These capillaries may arise from outside of the kidney and connect to the internal vasculature like the arteries.

### Loss of Ntn1 impairs recruitment of arterial smooth muscle and causes premature SMC differentiation at the kidney surface

**Fig. 4.**
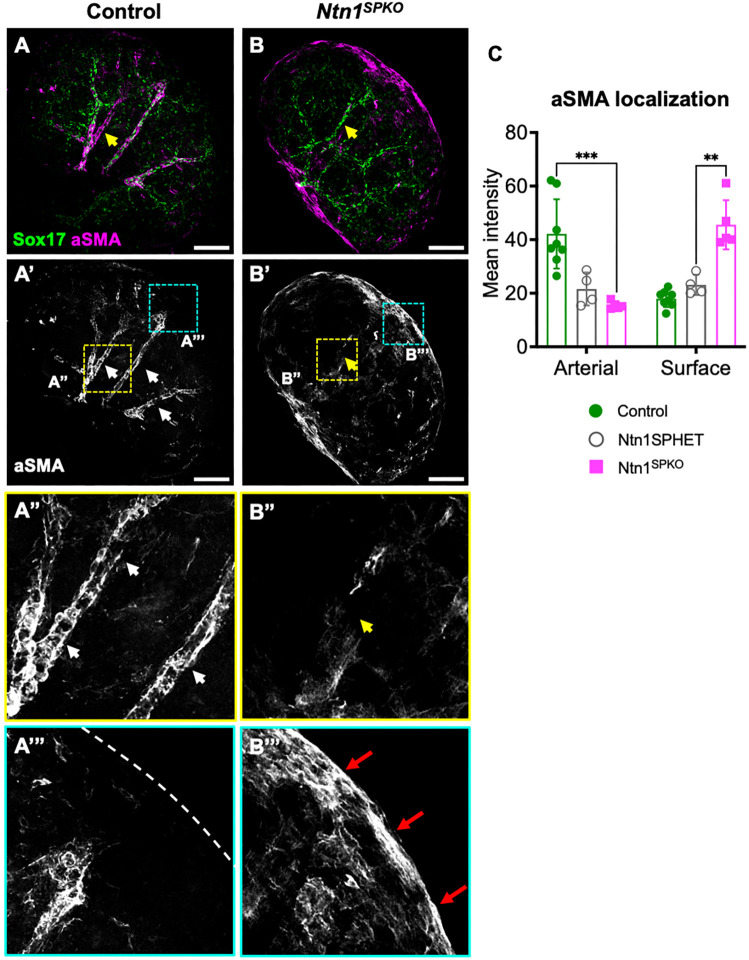
***Ntn1^SPKO^* kidneys exhibit decreased arterial smooth muscle and ectopic smooth muscle accumulation at the kidney surface at E13.5.** (A,B) Whole-mount immunofluorescence (WMIF) of arterial marker Sox17 and smooth muscle marker αSMA in E13.5 kidneys. (A′,B′) Single channel images for αSMA. (A″,B″) Single channel inset of arteries. White arrows, arterial vSMCs; yellow arrows, artery without smooth muscle in mutant. (A‴-B‴) Single channel inset of surface of kidney. Red arrows, ectopic smooth muscle in mutants. Dashed line in control indicates kidney surface. (C) Quantification of arterial and surface smooth muscle coverage, measured by mean pixel intensity within 25 µm or 40 µm of the Sox17^+^ arteries or kidney surface, respectively. *n*=5, ***P=0.000881 (arterial), **P=0.0027 (surface). Comparison of *Ntn1^SPHET^* (*Foxd1^GC/+^;Ntn1^f^*^/+^) and *Ntn1^SPKO^* trends towards significance (*P*=0.0662). Each *n*=1 embryo, multiple litters represented per experiment. Bar graphs show mean±s.e.m. *P*-values calculated by unpaired two-tailed *t*-test. Scale bars: 100 µm (A,B).

Recruitment of mural cells, including vSMCs, is an important step in arterial development (Gaengel et al., 2009) and arterial branching (Hurtado et al., 2015). Because mural cells derive from cortical SPs (i.e. the Foxd1^+^ lineage) in the kidney (Humphreys et al., 2010), we characterized mural cell recruitment without Ntn1. We assessed arterial vSMC coverage in E13.5 kidneys using WMIF for αSMA and Sox17 (Fig. 4A-C). Control arteries were covered with αSMA^+^ cells along their length, whereas *Ntn1^SPKO^* kidneys showed little arterial vSMC coverage (Fig. 4A′-B″). Instead, we found αSMA^+^ cells accumulated on the kidney surface, where cortical SPs reside (Fig. 4A‴,B‴), indicating that some progenitors had undergone ectopic differentiation to smooth muscle near their origin rather than at the arteries.

To confirm that ectopic SMCs are derived from the same progenitors that contribute to normal vSMCs, we crossed in a fluorescent reporter allele (LSL-tdTomato) to trace the *Foxd1^GC^* lineage in mutants and Cre^+^ controls. At E13.5, immunostaining for tdTomato showed that ectopic SMCs and normal arterial vSMCs (in controls) were indeed derived from Foxd1-expressing SPs (Fig. S4A-B″).

At 15.5, WMIF for αSMA showed significantly decreased arterial length covered with smooth muscle in mutants (Fig. S4C-E). In addition, examination of αSMA staining showed that first order vessels (relative to the initial renal artery and excluding bifurcations, green arrowheads) showed no significant difference in fluorescence intensity, but second order vessels (branches off first order branches, including arcuate arteries, yellow arrowheads) showed significantly lower staining intensity in *Ntn1^SPKO^* (Fig. S4C,D,F-H′). We noted that, when present, penetrating arteries had stronger αSMA staining that decreased as they entered the kidney. Intriguingly, ectopic αSMA^+^ cells remained present at the kidney surface at E15.5 (Fig. S4G″,H″, yellow arrowheads), and did not resolve to contribute to vascular smooth muscle. Because decreased vSMC coverage in *Ntn1^SPKO^* kidneys persists into adulthood, along with decreased arterial tree branching (see also figure 5 in Honeycutt et al., 2023), we posit that decreases in ramified branching are secondary to vSMC dysregulation.

### NG2^+^ stromal cell identity and Ntn1 requirement

**Fig. 5.**
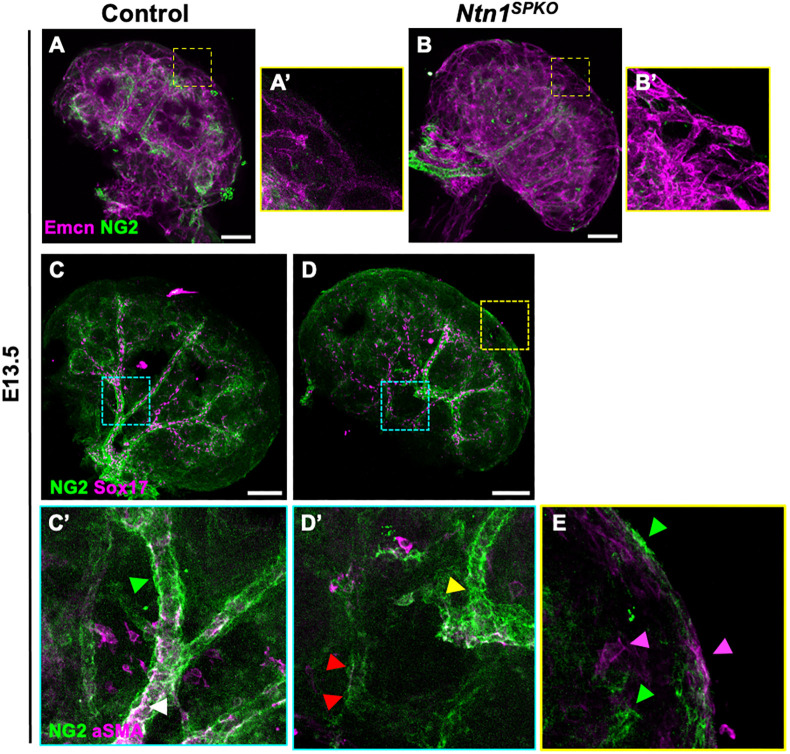
**Loss of Ntn1 from the kidney stroma results in ectopic NG2^+^ cells at the kidney surface and decreased investment along intra-renal arteries.** (A,B) Whole-mount immunofluorescence (WMIF) for NG2 and Emcn in E13.5 kidneys. (A′,B′) Magnifications of boxed areas show capillaries beyond capillary plexus in nephrogenic zone. (C,D) WMIF for NG2 and Sox17 in E13.5 kidneys. (C′,D′) Magnification of cyan boxed areas showing intrarenal arteries with co-stains for αSMA. White arrowhead, smooth muscle cells co-staining for NG2 and αSMA; green arrowhead, mural cells expressing only NG2; red arrowheads, NG2^+^ cells around intrarenal arteries in mutants; yellow arrowhead, penetrating artery with strong NG2 staining. (E) Magnification of yellow boxed area showing surface of *Ntn1^SPKO^* kidney. Both NG2^+^ cells (green arrowheads) and αSMA^+^ cells (magenta arrowheads) are seen at the surface, but there is a lack of overlap in expression. Scale bars: 100 µm (A-D).

We interrogated whether distribution of capillary-associated pericytes (also stromal-derived) is affected without Ntn1 using WMIF for NG2 (Cspg4), a commonly used marker of pericytes, and Emcn in E13.5 kidneys. Surprisingly, NG2 staining was not present around cortical capillaries in either control or *Ntn1^SPKO^* kidneys (Fig. 5A-B′). Instead, we found that NG2^+^ cells invested around arteries in both control and mutant kidneys (Fig. 5C-D′). Co-staining for αSMA showed that inner layers of SMCs expressed NG2 (Fig. 5C′, white arrowhead), but outer layers of mural cells expressed only NG2, with little to no αSMA (Fig. 5C′, green arrowhead). NG2^+^ mural cells were still able to invest arteries in *Ntn1^SPKO^* kidneys but appeared markedly reduced (Fig. 5D′, red arrowheads). Notably, penetrating arteries had more NG2 staining, again suggesting that these vessels may have more mural cell investment from early stages (Fig. 5D′, yellow arrowhead). However, NG2^+^ cells were also visible at the surface of the kidney (Fig. 5E, green arrowheads). Interestingly, co-staining with αSMA (magenta arrowheads) showed incomplete colocalization with ectopic smooth muscle at the surface. These data suggest that NG2^+^ cells around arteries in *Ntn1^SPKO^* kidneys are distinct from vSMCs. These cells are also mislocalized without Ntn1, but are not completely gone from arteries.

At E18.5, NG2^+^ pericytes associated normally with capillaries in both control and *Ntn1^SPKO^* kidneys, both in kidney cortex glomeruli and peritubular capillaries (Fig. S5A,B), and in the medulla (Fig. S5C,D) around the descending vasa recta. We note that NG2 IF at this stage marks true pericytes that interface directly with the endothelium. It is unknown whether these pericytes are related to NG2^+^/SMA^−^ cells surrounding arteries earlier in developmental time. Regardless, these data suggest that the role of Ntn1 in early kidney development is primarily on smooth muscle progenitors, with milder effects on other mural cell types such as pericytes.

### Loss of Ntn1 results in transcriptional dysregulation of vascular smooth muscle differentiation via loss of Klf4

**Fig. 6.**
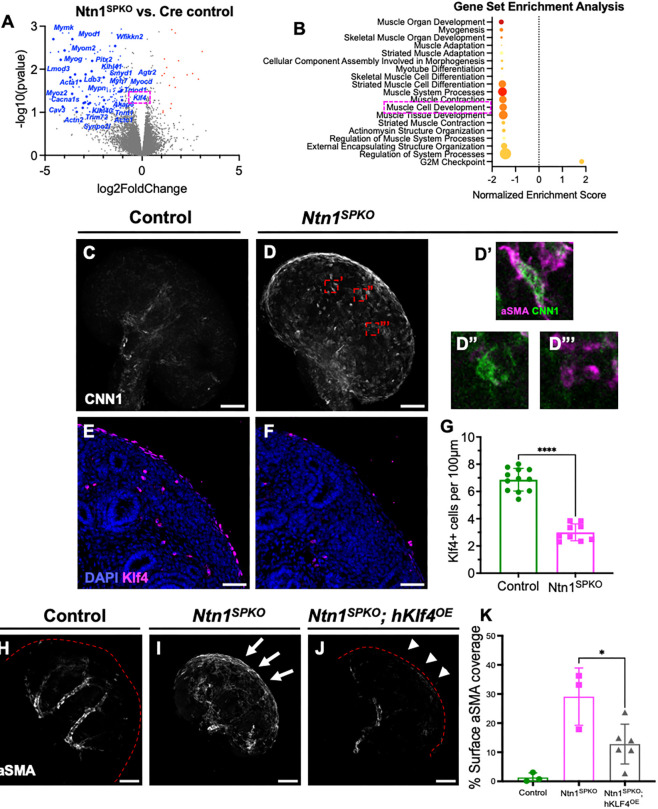
***Ntn1^SPKO^* kidneys display loss of Klf4 at E13.5 in stromal progenitors and premature differentiation into smooth muscle at the kidney cortex.** (A) Volcano plot of differentially expressed genes. Red and blue dots indicate genes with at least 2-fold up- or downregulation and false discovery rate <0.1, respectively. Annotated genes are from GO:005501 (muscle cell development) or have known roles in muscle development. (B) Gene set enrichment analysis of differentially expressed genes. Size of dot correlates to the size of the gene set. (C,D) Whole-mount immunofluorescence (WMIF) for CNN1 in E13.5 kidneys, showing localization of differentiated smooth muscle. (D′-D‴) Magnification of red boxed areas showing single contractile cells at the kidney surface showing heterogeneity of marker expression including αSMA^+^/CNN^+^ double-positive cells and single-positive cells. (E,F) Immunofluorescence of Klf4 protein on E13.5 kidney sections in cortical stromal progenitors. (G) Quantification of nuclear Klf4^+^ foci along the surface of E13.5 kidneys (*n*=9, ****P<0.0001). (H-J) WMIF for αSMA of E13.5 kidneys from control, *Ntn1^SPKO^* or hKlf4 overexpression in *Ntn1^SPKO^* background. Kidney surface is outlined (red dashed line) where not visible. White arrows, ectopic smooth muscle coverage; white arrowheads, less smooth muscle upon overexpression of Klf4. (K) Quantification of percentage of surface covered by smooth muscle, measured by percentage of pixel area in thresholded maximum intensity projections (*n*=3, *P=0.0215). Arterial smooth muscle was discounted in quantifications by manually removing pixels from individual slices. Each *n*=1 embryo, multiple litters represented per experiment. Bar graphs show mean±s.e.m. *P*-values calculated by unpaired two-tailed *t*-test. Scale bars: 100 µm (C,D,H-J); 50 µm (E,F).

To determine the mechanisms by which loss of Ntn1 affects developing kidneys, we isolated RNA from E13.5 *Foxd1^GC^* control and *Ntn1^SPKO^* kidneys and performed whole transcriptome bulk RNA-seq, followed by differential gene expression analysis. When compared with controls, 193 genes were significantly downregulated (*P*<0.1), and 26 genes were significantly upregulated in *Ntn1^SPKO^* kidneys (Fig. 6A). Gene set enrichment analysis showed downregulation of developmental pathways without Ntn1, especially those involved in muscle cell differentiation (Fig. 6B). This provided further evidence that αSMA^+^ cells at the kidney surface are prematurely differentiated vSMCs, rather than an unspecific fibrotic process such as myofibroblasts. We performed WMIF for CNN1, a regulator of smooth muscle contractility and marker of differentiated SMCs, and found that it was upregulated at the kidney surface (Fig. 6C,D). Many ectopic cells in mutant kidneys co-expressed CNN1 and αSMA (Fig. 6D′), but the presence of cells only expressing one marker (Fig. 6D′,D‴) suggested a heterogeneous response to the lack of Ntn1. Notably, common gene sets associated with vessel growth and patterning were not dysregulated at this stage, suggesting that the effects of Ntn1 on arterial branching may indeed be secondary to its regulation of smooth muscle differentiation.

To understand the molecular mechanism of SMC regulation by Ntn1, we looked for key upstream regulators in our RNA-seq data. The transcription factor Klf4 was downregulated without Ntn1. Klf4 is an evolutionarily conserved zinc-finger transcription factor required to maintain vSMCs in a mesenchymal state (Yap et al., 2021). Klf4 protein prevents binding of the muscle transcription factor myocardin and its coactivators SRF and MRTF-A/B to CarG boxes (Liu et al., 2005). *In situ* hybridization showed expression of *Klf4* in cortical SPs in E13.5 kidneys (Fig. S6A′), but not at earlier and later time points (Fig. S6A,A″,A‴), suggesting tightly controlled expression. Immunofluorescence at E13.5 showed Klf4 protein in the outermost, peripheral Foxd1^+^ SPs (Fig. 6C; Fig. S6B-B‴).

We and others have identified previously uncharacterized molecular heterogeneity in the developing renal stroma using scRNA-seq (Combes et al., 2019; England et al., 2020). This is further evidenced here by stronger Klf4 staining in a subset of Foxd1^+^ progenitors (Fig. S6B). To validate SP heterogeneity by RNA expression, we performed RNAscope expression analysis on E13.5 kidneys. In agreement with *in situ* hybridization, both *Ntn1* and *Klf4* are expressed in the cortical stroma (Fig. S6C), with little overlap of *Ntn1* and *Six2* expression in NPCs, or cytokeratin (CK) protein in the Ub (Fig. S6C′). Comparing *Klf4* and *Ntn1* expression (Fig. S6C″), we note that *Klf4* is expressed strongly on the outermost rim of SPs (Fig. S6C″, green arrowhead), whereas *Ntn1* is expressed further in (Fig. S6C″, magenta arrowhead). There is significant overlap of expression (green/magenta arrowhead), but expression patterns remain distinct. *Klf4* is also expressed at low levels within the kidney in *Pecam1^+^* endothelial cells (Fig. S6C‴), and is known to mediate flow response in the arterial endothelium (Chong et al., 2011). However, immunofluorescence of Klf4 in endothelial cells at E13.5 was low, suggesting a primary role in cortical stromal cells. Given the known role of Klf4 in SMCs and their origin within the Foxd1^+^ lineage, we infer that Klf4^+^ SP cells are precursors to renal vSMCs.

To validate Klf4 downregulation seen by RNA-seq, we performed immunostaining for Klf4 on E13.5 kidneys. In *Ntn1^SPKO^* kidneys, Klf4 was significantly reduced in the SPs (Fig. 6E-G). Given its reported role in SMC differentiation, this suggested that loss of Klf4 in SPs resulted in ectopic differentiation to contractile smooth muscle at the kidney surface.

To assess the dynamics of Ntn1 and Klf4 expression, we performed immunostaining for Klf4 at E15.5 and E18.5 (Fig. S6D-G). By E15.5, Klf4 was not expressed in all SPs in control kidneys, and was restricted to the outermost cells at the kidney periphery (Fig. S6D, inset). These outermost Klf4^+^ cells were not decreased in mutants, suggesting that Ntn1 affects Klf4^+^ SPs at E13.5 but not those remaining at E15.5. Starting at E15.5, Klf4 became expressed in arterial and glomerular endothelial cells, which were not affected by loss of Ntn1 (Fig.S6D-G). This indicates tight temporal regulation of Klf4 expression and protein within SPs.

To show that Klf4 is sufficient to prevent ectopic smooth muscle in SPs, we used a Cre-driven doxycycline-inducible system to selectively overexpress Klf4 in Foxd1-derived stromal cells in the *Ntn1^SPKO^* background. Upon expression of Klf4 in SPs, we observed significantly less ectopic SMC differentiation at the kidney surface by αSMA WMIF (Fig. 6H-K). Klf4 overexpression did not, however, restore smooth muscle coverage at the arteries, likely due to an inability of vSMC progenitors to differentiate due to Klf4 overexpression. Klf4 overexpression also did not rescue gross arterial patterning defects (Fig. S6H-J), nor did it restore kidney size in the *Ntn1^SPKO^* background (Fig. S6K), suggesting these effects of Ntn1 loss were independent of Klf4.

**Fig. 7.**
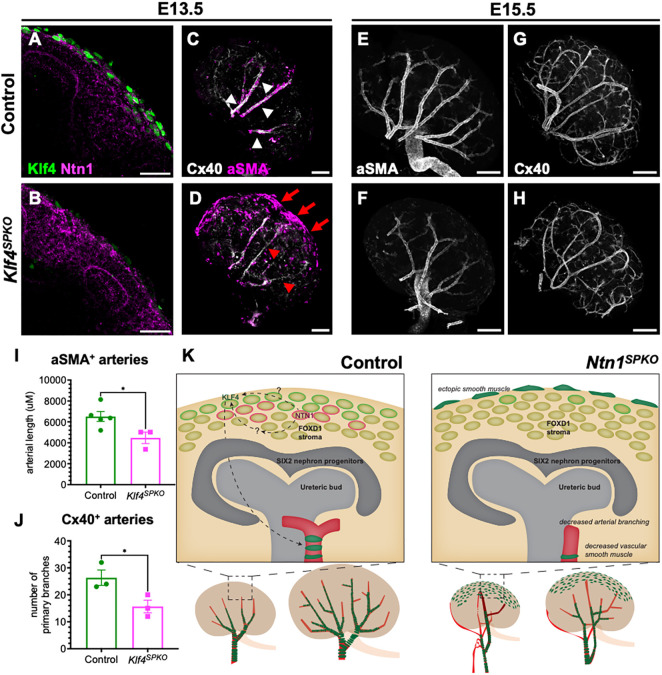
**Loss of Klf4 in the stromal progenitors results in ectopic differentiation of smooth muscle progenitors and decreased smooth muscle-induced arterial branching.** (A,B) Immunofluorescence of Klf4 and Ntn1 protein on E13.5 control and *Klf4^SPKO^* kidney sections. (C,D) Whole-mount immunofluorescence (WMIF) of E13.5 *Klf4^SPKO^* kidneys for Cx40 and αSMA to show localization of arterial smooth muscle cells. White arrowheads, smooth muscle covered arteries; red arrows, accumulation of smooth muscle on the surface; red arrowheads, arterial smooth muscle. (E,F) WMIF for αSMA on E15.5 kidneys. (G,H) WMIF for Cx40 on E15.5 kidneys to show branching of arteries in mutants. (I) Quantification of αSMA^+^ arterial length at E15.5 (*n*=3, *P=0.0342). (J) Quantification of primary branches in E15.5 kidneys (*n*=3, *P=0.0442). (K) Model of dysfunction in *Ntn1^SPKO^* kidneys, showing potential mechanisms of action (control on left, *Ntn1^SPKO^* on right). Each *n*=1 embryo, multiple litters represented per experiment. Bar graphs show mean±s.e.m. *P*-values calculated by unpaired two-tailed *t*-test. Scale bars: 50 µm (A,B); 100 µm (C,D); 200 µm (E-H).

We then ablated *Klf4* using the *Foxd1^GC^* driver line (*Klf4^SPKO^*) to test its necessity in preventing differentiation. We confirmed loss of Klf4, but not Ntn1, in E13.5 kidneys by IF (Fig. 7A,B). Loss of *Klf4* from SPs resulted in SMC accumulation at the kidney surface, similar to the *Ntn1^SPKO^* (Fig. 7C,D; Fig. S7E). Notably, loss of stromal Klf4 caused less severe defects in early (proximal) renal artery patterning than *Ntn1^SPKO^*, as there was no significant difference in Cx40^+^ arterial length within E13.5 *Klf4^SPKO^* kidneys (Fig. S7A,B,F), and arteries were also perfusable by IB4 (Fig. S7C,D). These results suggest that Ntn1 affects early gross renal artery patterning by Klf4-independent mechanisms.

At E15.5, arteries in *Klf4^SPKO^* kidneys exhibited decreased smooth muscle coverage, as measured by total length of smooth muscle covered arteries in αSMA WMIF (Fig. 7E,F,I). To verify that loss of stromal Klf4 did not affect overall arterial development, we measured total arterial length using Cx40 WMIF at E15.5 and found no significant difference (Fig. 7G,H; Fig. S7G).

Both we and Honeycutt et al. (2023) find that loss of Ntn1 results in decreased branching of the renal arterial tree, potentially downstream of decreased smooth muscle coverage. To test whether loss of smooth muscle coverage alone is sufficient to delay branching, we counted primary branches in E15.5 *Klf4^SPKO^* kidneys using Cx40 WMIF. Similar to the *Ntn1^SPKO^*, there were fewer branches of main arteries without Klf4 (Fig. 7J). However, in contrast to loss of Ntn1 alone, E15.5 *Klf4^SPKO^* kidneys were not smaller than controls (Fig. S7H), suggesting that the decrease in kidney size upon loss of Ntn1 is not due to excess contractile smooth muscle or decreased arterial branching, as these defects are found in both *Klf4^SPKO^* and *Ntn1^SPKO^* kidneys.

## DISCUSSION

SPs have been implicated in patterning of the kidney vasculature, but the molecular basis for this role has been unclear. In this study, we show that Ntn1 is required for early development and patterning of the kidney vasculature and for proper localization of vascular mural cells. We show that profound defects in arterial patterning occur upon loss of Ntn1 expression from Foxd1^+^ SPs. In addition, we show that SPs at the periphery of *Ntn1^SPKO^* kidneys prematurely and ectopically differentiate into contractile smooth muscle, resulting in decreased arterial vSMC coverage. We also find that the transcription factor Klf4 is significantly downregulated upon loss of Ntn1, and that stromal loss of Klf4 leads to similar dysregulation of mural cell localization. Finally, we show that loss of Klf4 from SPs results in decreases in ramified arterial branching, but not in proximal artery patterning defects as observed in *Ntn1^SPKO^* kidneys. Together, these data suggest that the Ntn1-Klf4 axis is a previously unreported mediator of stromal crosstalk that regulates mural cell differentiation and vascular patterning within the developing kidney, underscoring the idea of an instructive and heterogeneous kidney stroma.

### Ntn1-mediated crosstalk in the nephrogenic zone

Using scRNA-seq and *in situ* hybridization, we and others identified Ntn1 expression in the cortical SPs during renal development (Combes et al., 2019; England et al., 2020; Honeycutt et al., 2023). Our data show that Ntn1 protein and RNA expression begins early during nephrogenesis. Expression is restricted to the Foxd1^+^ SPs at E13.5, matching our previous characterization of E18.5 stromal heterogeneity. Ntn1 protein, by contrast, is found diffusely surrounding both stromal and epithelial cells in the nephrogenic zone. The presence of protein in and around cells without transcripts shows that Ntn1 is secreted from SPs into the cortex. In particular, Ntn1 protein appears to be enriched at the terminal Ub tips. It is possible that the Ub serves as a ligand sink, constraining Ntn1 to the cortical nephrogenic zone throughout development. However, given the decrease in Ub branching in *Ntn1^SPKO^* kidneys, a direct signaling role cannot be excluded.

By P3, Ntn1 protein is no longer detectable in the kidney cortex, coinciding approximately with the cessation of nephrogenesis. This tight spatiotemporal restriction of Ntn1 RNA and protein is striking and suggests a specialized role in regulating kidney development. Given its known role as a guidance cue for endothelial cells, we sought to test whether Ntn1 regulates the formation and patterning of renal blood vessels.

### Ntn1 is essential to normal vascular patterning and arteriogenesis

Loss of Ntn1 from SPs results in profound defects of renal blood vessel development. One of the most striking defects in *Ntn1^SPKO^* kidneys is abnormal patterning of the primary renal artery, which we show leads to decreased blood flow into the early kidney. Normally, the renal artery splits into two branches shortly before entering the kidney at opposite sides of the hilum. Large arteries continue to branch iteratively within the developing kidney to reach the cortical glomeruli.

We find that the stereotyped pattern of arterial connection of the kidneys to the aorta is lost in Ntn1 mutants. These defects are accompanied by a decrease in perfusion and flow response within early arteries. *Ntn1^SPKO^* mutants exhibit excessive branching of the renal artery between the aorta and kidney, suggesting either an increase in angiogenic activity or a failure of vessels to remodel into a singular renal artery. This abnormal pattern is associated with arteries entering the kidney farther from the hilum, resulting in decreased perfusion and stunted growth of arteries within the kidney. We show this decreased blood flow both with IB4 perfusion into the embryonic vasculature and by flow-dependent Cx40 expression. How Ntn1 affects remodeling of arteries outside the kidney is unknown, but it is possible that Ntn1, secreted by SPs at the earliest stages of nephrogenesis into the angioblast-rich mesenchyme, regulates remodeling of the renal artery as it sprouts from the aorta.

The effects of early mispatterning in mutants is progressively minimized as the kidney grows and relative arterial distances from the hilum become closer to wild type. This is accompanied by increased perfusion and Cx40 expression in all arteries by E15.5, potentially due to increases in blood volume and pressures in the embryo or pruning of ectopic vessels that shunt blood away from the kidney.

Intriguingly, many of the gross patterning defects of the renal artery, such as contribution of multiple aortic branches to the renal vasculature, are reminiscent of the vasculature observed in the global Foxd1 deletion mouse model (Hum et al., 2014; Sequeira-Lopez et al., 2015). These observations raise the possibility that ectopic extra-kidney vessels are not solely due to loss of cortical Ntn1, but to the compound loss of Ntn1 and of the loss of one Foxd1 allele in the *Foxd1^GC^.*

### Ntn1 promotes ramified arterial branching by regulating smooth muscle differentiation

An important finding from our work and the work of others (Honeycutt et al., 2023) is decreased arterial tree branching within the kidney in the absence of Ntn1. It is possible that the aforementioned alterations in renal artery patterning, and resulting decreases in perfusion, have downstream effects on branching within the kidney. However, we propose that arterial branching is instead regulated by vascular smooth muscle coverage. Recruitment of mural cells such as vSMCs impacts vascular integrity and function, as well as vascular patterning (Armulik et al., 2011; Gaengel et al., 2009; Kemp et al., 2022; Orlich et al., 2022; Stratman et al., 2017). We find that arterial smooth muscle coverage is decreased without Ntn1, and Honeycutt et al. (2023) find that both smooth muscle coverage and arterial branching are affected well into adulthood, making it unlikely that the transient loss of flow during early nephrogenesis is the root cause. Furthermore, we find that Ntn1 promotes smooth muscle coverage of arteries by suppressing the differentiation of smooth muscle progenitors at the cortex via Klf4. Ablation of Klf4 from the renal stroma results in similarly decreased smooth muscle coverage and arterial branching, and alterations in early renal perfusion. This shows that ramified arterial branching is independent of early artery mispatterning downstream of loss of Ntn1. These findings are in line with previous characterizations of smooth muscle-related stromal mutants and their effects on arterial branching (Hurtado et al., 2015).

It is also possible that loss of direct Ntn1 signaling to arteries results in endothelial dysfunction and a failure to recruit smooth muscle. However, the presence of ectopic smooth muscle accumulated at the kidney surface suggests stromal-intrinsic regulation. Loss of Ntn1 results in decreased Klf4 in SPs, which is associated with ectopic expression of multiple contractile markers (such as CNN1 and αSMA) at the surface of the kidney. Klf4 has been shown to repress the activity of myocardin, along with its co-factor Srf, to prevent or reverse smooth muscle differentiation, and is often dysregulated during disease (Yap et al., 2021). We recently showed that stromal Srf was necessary for SMC differentiation in the kidney (Drake et al., 2022). Klf4 ablation results in ectopic accumulation of smooth muscle similar to loss of Ntn1, and overexpressing Klf4 in kidneys that lack Ntn1 results in less ectopic smooth muscle at the kidney surface, further evidencing the role of Klf4 role in regulating differentiation of kidney smooth muscle progenitors.

Notably, Klf4 overexpression does not restore arterial smooth muscle coverage at E13.5. We suspect that the level of overexpression achieved was enough to reduce ectopic differentiation at the surface, but likely too much to then allow proper downregulation of Klf4 at the arteries and differentiation to vSMCs. Alternatively, as the overexpression was performed in an *Ntn1^SPKO^* background, other Klf4-independent effects of Ntn1 ablation could result in a lack of signals to promote vSMC coverage. Regardless, these limitations prevented us from assessing whether restoring arterial smooth muscle would then rescue defects in branching. Altogether, these models provide insights into the role of Ntn1 in modulating smooth muscle coverage and arterial branching, as well as other effects likely not mediated through this pathway (see Fig. S7I).

### Heterogeneity in stromal progenitors and descendants

We have previously described the dramatic heterogeneity of the developing renal stroma at E18.5, focusing on zonation within the kidney and association with various epithelial components (England et al., 2020). Here, we find multiple instances of heterogeneity within the SPs and their mural cell derivatives in the developing kidney.

Most intriguingly, we observe different levels of Klf4 protein in Foxd1^+^ progenitors at E13.5. The Foxd1^+^ SPs were initially described as a multipotent and homogeneous population. However, cells at the kidney surface have more Klf4 protein. Expression of *Klf4* and *Ntn1* reinforces the heterogeneous nature of the SPs, as Ntn1 is not expressed in the outermost rim of Klf4^+^ cells, and conversely Klf4 is not expressed in Ntn1^+^ progenitors found further from the edge of the kidney. Given the known role of Klf4 in promoting a mesenchymal state in vSMCs (Majesky et al., 2017), we infer that Klf4^+^ cells may contain specific vSMC progenitors. Klf4 protein remains strong in the outermost cells of the kidney throughout development until E18.5. It is possible that these are cells of the kidney capsule, which is also part of the stromal lineage (Levinson et al., 2005). As Klf4 is involved in many processes and pluripotency in general (Takahashi and Yamanaka, 2006), co-expression with other genes such as *Myocd* might be required to specify a vSMC progenitor. Further studies with higher resolution expression data and new lineage tracing tools will be required to demonstrate specific lineages from progenitor to descendant, but here we present data suggesting a heterogeneous progenitor population.

We also describe novel relationships between different populations of mural cells surrounding blood vessels. The periarterial niche of mature vessels has been described as a heterogeneous mix of vSMCs, fibroblasts and other cell populations. Intriguingly, we find NG2, a marker of pericytes, expressed uniquely in some cells surrounding vSMCs in the periarterial niche. Whether these cells are related to pericytes found later in development around the capillary vasculature is an open question. As vSMCs also express NG2, it is possible that these simply represent immature smooth muscle, but analysis of kidneys lacking Ntn1 demonstrates investment of NG2^+^ cells without αSMA. In addition, markers of mature mural cells expressed at the surface of *Ntn1^SPKO^* kidneys display a heterogeneous response to lack of Ntn1, as coexpression of CNN1, αSMA and NG2 is incomplete. Again, higher resolution techniques will be required to understand the different cellular responses to lack of Ntn1.

A final manifestation of the heterogeneous stroma is the nature of the smooth muscle cells seen in later stages of *Ntn1^SPKO^* and *Klf4^SPKO^* kidneys. Despite continued presence of ectopic smooth muscle at the surface, arteries are covered with some (albeit significantly less) smooth muscle in these mutants. These vSMCs, while likely not derived from the original dedicated smooth muscle progenitors, could still originate from the cortical stroma as other cells may be able to compensate. Alternatively, the NG2^+^ cells around the arteries in *Ntn1^SPKO^* may transdifferentiate into smooth muscle and express αSMA. Another possible source is from outside of the kidney, such as the aortic smooth muscle or the Tbx18^+^ progenitors found in the ureter, which has been described to also give rise to vascular smooth muscle (Airik et al., 2006), but the contributions of these sources in control and mutant kidneys is still poorly understood. In support of one of these options, it appears that penetrating arteries have stronger αSMA staining that decreases as they travel into the kidney, suggesting that they bring in their own smooth muscle that could proliferate and ultimately cover the rest of the arterial tree, effectively rescuing the phenotype. No matter the source, this compensation demonstrates the resilience of the renal stroma and reflects its importance to kidney development.

### Downstream targets of Ntn1 signaling

We show that Ntn1 has many roles in the developing kidney, including regulating vascular patterning and mural cell differentiation, as well as epithelial morphogenesis (directly or indirectly). Given the expression of multiple known Ntn1 receptors, such as Unc5c, Neo1 and Unc5b, in various potential target cell types throughout the developing kidney, and Ntn1 restriction to the cortex throughout development, more in depth evaluation of Ntn1 signaling is needed. Moreover, some studies have suggested that Ntn1 has signaling functions that are independent of known receptors (Wilson et al., 2006), underscoring the difficulty of identifying responsible receptors in the kidney.

How Ntn1 affects Klf4 levels and SP differentiation is unclear. The presence of Ntn1 protein in the cortex, where Klf4^+^ cells are located, suggests a direct induction; however, there is no specific expression of a known Ntn1 receptor within the Klf4^+^ cells. Neo1 is expressed in the cortical stroma per *in situ* hybridization, but given the heterogeneity of the SPs that we observe, as well as the relatively ubiquitous expression of Neo1, further studies with higher resolution expression data will be required to investigate potential induction of Klf4 by Ntn1. Alternatively, Ntn1 could affect smooth muscle progenitors indirectly via a relay mechanism, either via other stromal cells or endothelial cells (see Fig. 7K). Interestingly, in other systems Klf4 has been shown to promote transcription of Ntn1 (Orgeur et al., 2018). This would represent an intriguing feedback mechanism whereby vSMC progenitors use Ntn1 to help control their differentiation state. We observe that Klf4 and Ntn1 are sometimes expressed in the same cells, but the most cortical SPs express only Klf4, and progenitors further from the surface express only Ntn1. In addition, loss of Klf4 from the Foxd1^+^ SPs alone does not result in loss of Ntn1 protein. If Klf4 does promote Ntn1 expression, therefore, it would likely be one of multiple factors regulating its expression, as Ntn1 expression is found within the kidney at least until E18.5, whereas Klf4 expression and protein abate by E15.5.

### Summary

In sum, these novel findings demonstrate how crucial the kidney stroma is to overall kidney vascular and epithelial development. Restricted expression of a single guidance cue, Ntn1, is required for proper differentiation of three important lineages within the kidney: the stroma itself (its derivatives, the mural cells), the vascular endothelium and the nephron epithelium. Mechanistically, we show that a key transcription factor Klf4 depends on Ntn1, and that loss of either impairs both mural cell investment and vascular development. Together, these findings underscore that the stroma is not merely supportive, but rather it is an essential partner in successful organogenesis.

## MATERIALS AND METHODS

### Mouse models

Experiments were performed in accordance with protocols approved by the University of Texas Southwestern Medical Center (UTSW) Institutional Animal Care and Use Committee. Timed matings were set up between females 7-9 weeks and males older than 7 weeks, and mating was ascertained by checking plugs, at which point the pregnancy was determined to be 0.5 days gestation. Dams were euthanized at desired time points and embryos were dissected for relevant tissues. Sex was not determined as developmental time points occur before sex differentiation. The following mouse lines were used: *Foxd1^GC^* [The Jackson Laboratory (JAX), 012463], Ntn1^flox^ (JAX, 028038), Klf4^flox^ [Mutant Mouse Resource and Research Centers (MMRRC), 029877-MU], LSL-rTTA (gift from Hao Zhu, UTSW, USA; JAX, 029617), tet-on-hKLF4 (gift from Mark Kahn, University of Pennsylvania, PA, USA; JAX, 019039) and B6.Cg-Gt(ROSA)26Sortm14(CAG-tdTomato)Hze/J (JAX, 007914). All mice were maintained on a primarily C57/b6 background, and *Ntn1^SPKO^* crosses were maintained in a pure background. Klf4 overexpression was achieved in the *Ntn1^SPKO^* background using tet-on-hKLF4 and LSL-rTTA by providing doxycycline hyclate (Sigma-Aldrich, 150 mg/ml) in drinking water along with 1% sucrose w/v from E11.5 until dissection (E13.5).

### Kidney and body length measurements

At E13.5, pictures of embryos were taken on a Excelis HDS camera (Accu-Scope) mounted to a dissecting microscope at 1.5× zoom (World Precision Instruments). Kidneys were dissected and images were taken on an Axios Imager.M2 (Zeiss) using a 5× objective. Kidney and embryo lengths were obtained by measuring the longest width using Fiji, and ratios were obtained by dividing kidney by embryo length. At P5, pups and kidneys were weighed using a scale, and kidneys were dried on a Kimwipe before weighing to eliminate water weight.

### IB4 injection of embryonic vasculature

IB4-Alexa Fluor 488 (Thermo Fisher Scientific) was prepared at 0.5 mg/ml and supplemented with 0.5% Evans Blue and injected with a 30 gauge needle into the livers of E13.5 or E15.5 embryos shortly after removing from the yolk sac. Successful injection was determined by observing Evans Blue incorporation into the head vasculature. Kidneys were placed directly into 4% paraformaldehyde (PFA)/PBS and fixed for 30 min at room temperature (RT) to preserve fluorescent signal. WMIF (see below) was performed to co-stain for arterial markers using other fluorescent channels, and kidneys were cleared and imaged per usual.

### Tissue preparation and immunofluorescence on sections

E13.5-P5 kidneys were dissected and fixed in 4% PFA/PBS overnight at 4°C, embedded in paraffin and sectioned as previously described (Daniel et al., 2018). Briefly, tissue was dehydrated to 100% ethanol, incubated in xylene twice for 10 min, a mixture of 1:1 xylene and paraplast for 10 min at 60°C, and replaced with paraplast three times at 60°C before embedding and sectioning at 10 µm with a Leica microtome onto SuperfrostPlus glass slides.

Immunofluorescence staining was performed as previously described. Briefly, paraffin sections were deparaffinized with xylene, rehydrated through an ethanol series into PBS, permeabilized with 0.3% PBS-Triton X-100 for 10 min with rotation, and treated with R-Buffer A or B (Electron Microscopy Sciences) for heat-mediated antigen retrieval of nuclear and cytoplasmic antigens, respectively, in a 2100 Retriever (Electron Microscopy Sciences). Slides were allowed to cool and then blocked in CAS Block (Invitrogen) for 2 h at RT. Slides were incubated in primary antibody overnight at 4°C, washed in PBS and incubated in secondary antibody for 1.5 h at RT. Slides were washed with PBS, treated with a blood lysis buffer (10 mM CuSO_4_, 50 mM NH_4_Cl, pH 5), incubated in deionized water for 5 min and washed with PBS again before mounting with DAPI-Fluoromount (Southern Biotech). Imaging was performed on a Nikon A1R confocal microscope. Antibodies used, concentrations and conditions are summarized in Table S1.

### Whole-mount immunofluorescence

E13.5-E18.5 kidneys were dissected as above, ensuring that all surrounding mesenchyme was removed to limit extra-renal fluorescent signal with certain antibodies. Tissue was dehydrated into 100% methanol and stored at 4°C until staining. For WMIF, kidneys were rehydrated into PBS, permeabilized with 1% (E13.5) or 3% (E15.5, E18.5) Triton X-100 in PBS for 3 h (E13.5, E15.5) or 6 h (E18.5). Kidneys were then blocked with CAS block for a minimum of 2 h and incubated with primary antibody overnight at 4°C. Tissue was washed with PBS for a minimum of 6 h-long washes and incubated in secondary antibody overnight at 4°C (see Table S1). Tissue was washed again at least six times, dehydrated into 100% methanol and cleared using 2:1 benzyl alcohol:benzyl benzoate (BABB) for at least 10 min. Cleared tissue was mounted in BABB into concavity slides (Electron Microscopy Sciences) and imaged using a LSM700 Zeiss confocal microscope.

### Image analysis

Linear adjustments, maximum intensity projections and cropping was performed in ImageJ. Manual removal of fluorescent signal was limited to ureteric smooth muscle and was performed slice by slice and indicated in the figure legends. αSMA signal attributable to ureteric smooth muscle was easily distinguished from arterial smooth muscle. Orthogonal and 3D projections were visualized using Imaris 9.0.0.2 or Imaris 10.0 software (Bitplane).

### Arterial length and branching measurements

To measure the length of intrarenal arteries, maximum intensity projections of WMIFs were created of the middle third of kidneys in order to ensure no confusion with arteries on the anterior or posterior surface of the kidney. Only kidneys that were oriented properly (lying flat with ureter orthogonal to the *z*-plane) were used. Arteries were traced manually in Fiji and total length was summed. Extrarenal arteries were measured using middle-third projections similarly. For αSMA and total artery length (Cx40) quantifications, the entire kidney was projected and arteries were traced manually.

Branching quantifications were performed on 3D projections in Imaris by manually counting the primary branches of the renal artery. Importantly, bifurcations and trifurcations were not counted, as these were counted as the same order in the arterial tree. These were defined as branch points where none of the branches continued in the same direction as the original artery and all branches were approximately the same diameter. In addition, arteries directly feeding into glomeruli were not counted, as these branches are not part of the ramified tree-like structure and can arise from any order branch, depending on the proximity to the cortex and developing nephrons. In addition, it is likely that the glomerulus secretes angiogenic factors to promote branching, which is unrelated to the smooth muscle-dependent ramification we aimed to capture.

### *In situ* hybridization

Ntn1, Unc5b, Neo1 and Unc5c coding sequences were obtained from Dharmarcon (Ntn1, BC029161; Unc5b, BC048162; Neo1, BC054540; Unc5c, BC115772) and digoxigenin-labeled RNA probes were synthesized as previously described (Daniel et al., 2018). Briefly, plasmids were linearized and purified by phenol:chloroform extraction. Probes were synthesized at 37°C for 2-4 h in digoxigenin-synthesis reaction mixture with T7 RNA polymerase (Roche). After synthesis, DNA was eliminated with DNase I (Promega) and RNA probes were purified using Micro Bio-Spin columns (Bio-Rad). Final concentration for hybridization was 1 µg/ml and was achieved by diluting in pre-hybridization buffer. *In situ* hybridization was performed on paraffin-embedded sections as previously described (Daniel et al., 2018). Briefly, slides were deparaffinized in xylene and rehydrated, after which they were treated with 15 µg/ml Proteinase K for 15 min and fixed in 4% PFA. Slides were incubated in prewarmed pre-hybridization buffer for 1 h followed by overnight incubation with diluted RNA probe at 65°C. Slides were then washed in 0.2× SSC and maleic acid buffer with 0.5% Tween-20 (MBST) before blocking with 2% blocking solution (Roche) for at least 1 h at RT. Slides were then incubated overnight with Anti-Dig alkaline phosphatase-conjugated antibody (Roche, 11093274910, 1:4000) at 4°C. Slides were washed with MBST and sodium chloride/Tris base/magnesium chloride/Tween-20 (NTMT) and incubated with BM Purple (Roche) for colorimetric reaction. Slides were post-fixed in 4% PFA and mounted in Permount (Thermo Fisher Scientific). Slides were imaged using a Zeiss Axiovert 200M scope.

### HiPlex RNAscope fluorescent *in situ* hybridization

HiPlex RNAscope probes were obtained from Advanced Cell Diagnostics: Mm-Ntn1-T1 (407621-T1), Mm-Klf4-T2 (426711-T2), Mm-Foxd1-T3 (495501-T3), Mm-Six2-T10 (50011-T10), Mm-Pecam1-T11 (316721-T11). E13.5 kidneys were dissected as above and fresh frozen in OCT compound (Scigen) with liquid nitrogen. Kidneys were sectioned with a cryostat at 10 µm and RNAscope fluorescent HiPlex hybridization was performed following the manufacturer's instructions using the HiPlex v2 reagent kit (324419). Images were acquired on a Nikon A1R confocal microscope. IF was performed on the same section as described above by applying CAS-block to the sections after final cleaving steps of the HiPlex assay. Images were analyzed in Fiji and registered manually.

### Western blot

E13.5 kidneys were dissected as above. Protein was extracted in RIPA buffer for 30 min and quantified by BCA assay (Thermo Fisher Scientific). Then, 10 µg protein was run on a polyacrylamide gel, blocked with blocking buffer (Bio-Rad), blotted with primary antibody overnight at 4°C, washed with Tris-buffered saline with 0.1% Tween-20 (TBST), incubated in secondary antibody, and developed using ECL and X-ray film. Densitometry analysis was performed on scanned images using Fiji. Antibodies used and concentrations are detailed in Table S1.

### Glomerular count (acid maceration)

HCl maceration of whole kidneys was performed according to MacKay et al. (1987). Briefly, kidneys were isolated from 6-week-old control and *Ntn1^SPKO^* mice, decapsulated, roughly chopped with a razor blade and incubated in 5 ml 6M HCl per kidney for 90 min. Every 30 min, the kidneys were pipetted up and down to further disrupt the kidneys. Digested kidneys were diluted with 5 volumes (25 ml) of distilled water and incubated at 4°C overnight. For counting, 1 ml of macerate was pipetted into a cell culture dish with grid lines and glomeruli were counted.

### RNA-seq, differential gene analysis and data availability

RNA was extracted from whole E13.5 embryonic kidneys with an RNeasy Plus Micro kit (Qiagen). Concentrations and quality of RNA were assessed before sequencing (UTSW Genomics Core). Transcript abundance was estimated without aligning reads using Salmon (Patro et al., 2017) against an index of coding sequences from the Ensembl GRCm38 assembly. Transcript-level abundance was imported and count and offset matrices generated using the tximport R/Bioconductor package. Differential expression analysis was performed using the DESeq2 R/Bioconductor package (Love et al., 2014). Genesets were downloaded from MSigDB (Liberzon et al., 2011; Subramanian et al., 2005) using the msigdbr R package. The R/Bioconductor package fgsea was used to carry out gene set enrichment analysis. RNA-seq data have been deposited in the Gene Expression Omnibus (GEO) under the series accession number GSE242508.

### Data analysis and visualization

All data were plotted and graphs were generated in PRISM 9 XML. No statistical methods were used to predetermine sample sizes. All quantifications were carried out with at least *n*=3 embryos from two separate litters. No samples were excluded unless mentioned (see ‘Arterial length and branching measurements’, where only images oriented correctly were analyzed). No experiments were randomized for animal studies, and investigators were aware of group allocation for outcome assessment, except for glomerular counting, where counts were performed before genotyping. All comparisons were carried using the parametric unpaired two-tailed Student's *t*-test unless otherwise mentioned in figure captions. ns, *P*>0.05 (not significant); *P<0.05; **P<0.01; ***P<0.001; ****P<0.0001. Linear manipulations of images were carried out using ImageJ. Figures and models were made using Microsoft Powerpoint, BioRender.com and Adobe Illustrator.

## Supplementary Material

10.1242/develop.201884_sup1 Supplementary information
