# Supplementary material for: Stromal netrin 1 coordinates renal arteriogenesis and mural cell differentiation
Source: Development. 2023 Nov 24;150(22):dev201884. doi: 10.1242/dev.201884 (PMC10690105; doi:10.1242/dev.201884)
Supplement: Supplementary information [file develop-150-201884-s1.pdf]

Fig. S1. Netrin-1 mRNA and protein expression by cortical stromal progenitors during midgestation kidney development.

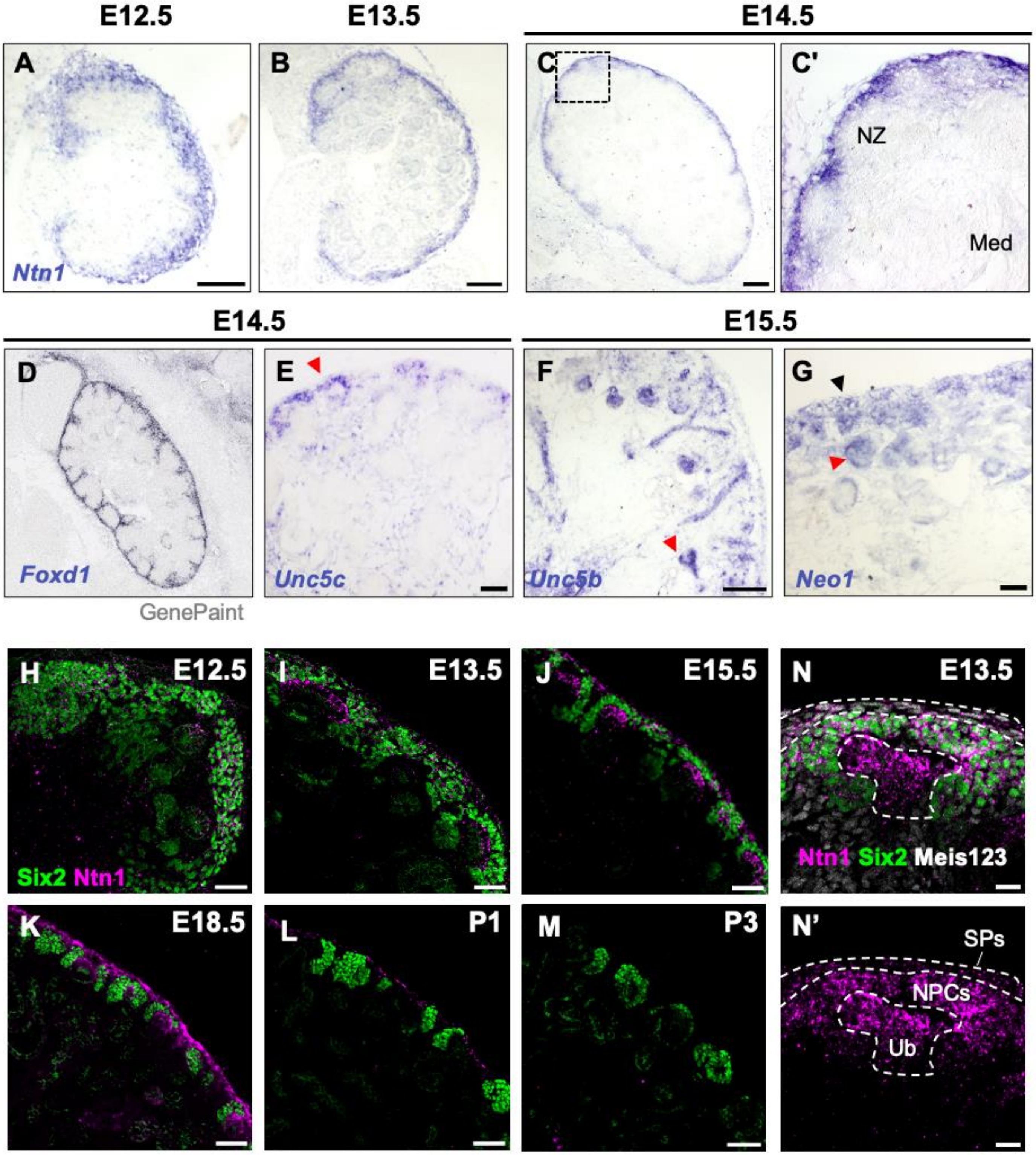

Fig. S1. Netrin-1 mRNA and protein expression by cortical stromal progenitors during midgestation kidney development. (A-C) *in situ* hybridization for *Ntn1* in E12.5-E14.5 kidneys, showing cortical expression that overlaps with previously characterized *Foxd1* expression. Inset (C') shows restriction to the stromal progenitors, while no expression in the nephrogenic zone (NZ) or the medullary region (Med). (D) Publicly available E14.5 *in situ* hybridization (Genepaint) for *Foxd1*, showing regional expression similar to *Ntn1*. (E-G) *In situ* hybridization against netrin-1 receptors *Unc5c*, *Unc5b*, and *Neo1*. Red arrowheads indicate expression in nephron progenitor cells, glomerular vessels, and ureteric epithelia, respectively. Black arrowhead indicates stromal expression of *Neo1*. (H-M) Immunofluorescence (IF) staining for Ntn1 in E12.5-P3 kidneys (green), showing restriction to the outer cortical region throughout development near nephron progenitor cells (NPCs) marked by Six2 (magenta). Ntn1 levels decrease rapidly after birth and are undetectable by IF at P3. (N,N') E13.5 IF for Ntn1 with costains for Six2 and Meis1/2/3, showing distribution of Ntn1 protein in early nephrogenesis primarily within the NZ. Dotted lines outline borders of Foxd1^{+} stromal progenitors (SPs), Six2 NPCs, and the ureteric bud tip (Ub). Scale bars: 200µm (A), 100µm (B-F), 50µm (H-K), 20µm (N).

Development • Supplementary information

Development: doi:10.1242/dev.201884: Supplementary information

Fig. S2. Loss of stromal netrin-1 in the kidney results in decreased kidney size, delayed nephrogenesis, and decreased number of glomeruli.

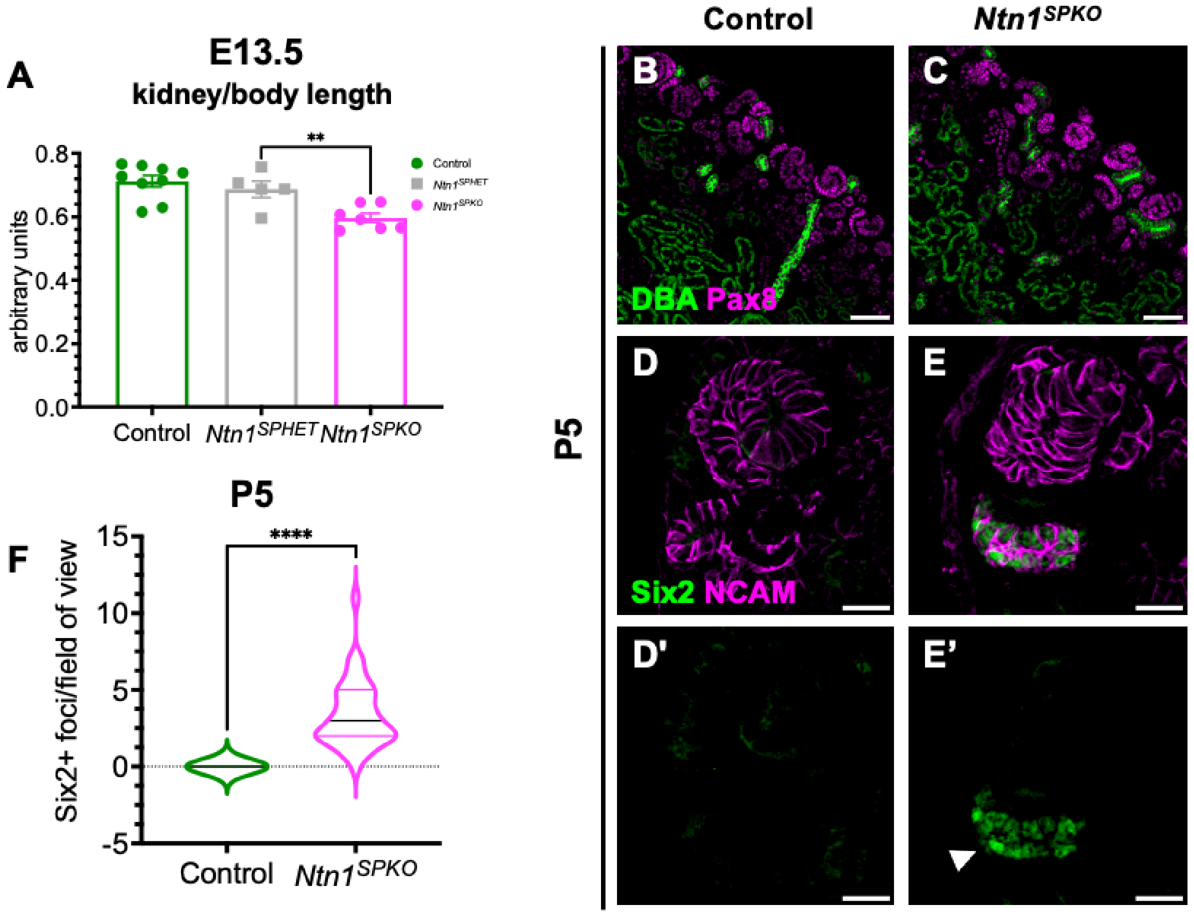

P5

G

Body weight (g) Control	Body weight (g) Ntn1SPKO	Kidney weight (g) Control	Kidney weight (g) Ntn1SPKO	k/b ratio Control	k/b ratio Ntn1SPKO
2.95	2.72	0.01635	0.01355	0.55	0.50
2.7	2.72	0.01305	0.01105	0.48	0.41
2.61	3.14	0.01505	0.01255	0.58	0.40
3.04	2.73	0.01335	0.01185	0.44	0.43
3.13		0.01645		0.53	
2.95		0.0149		0.51	
3.22		0.0186		0.58	
3.12		0.01835		0.59	
3.09		0.0171		0.55	
3.11		0.01805		0.58	
2.79		0.015		0.54	
2.7		0.0131		0.49	
3.25		0.0172		0.53	

P40

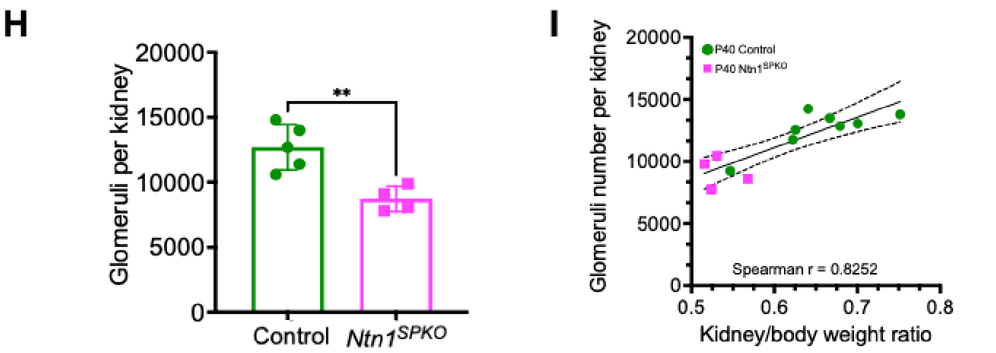

Development • Supplementary information

Fig. S2. Loss of stromal netrin-1 in the kidney results in decreased kidney size, delayed nephrogenesis, and decreased number of glomeruli. (A) Kidney/embryo length for E13.5 control and *Ntn1*^{SPKO} embryos, showing decreased kidney length when controlled for overall embryo development (n=7, p=0.0086) (B-C) IF staining of P5 control and *Ntn1*^{SPKO} kidneys for DBA and Pax8, showing relatively normal morphology of collecting duct epithelia and nephrogenic epithelial structures, respectively. (D-E) IF staining for Six2 and NCAM, a marker of the renal vesicle stage of nephrogenesis, showing perdurance of nuclear Six2 signal and prolongation NPC presence in *Ntn1*^{SPKO} kidneys. (F) Quantification of Six2^{+} nuclear foci number in control vs mutant kidneys, showing significant increase in *Ntn1*^{SPKO} kidneys (n=3, p<0.0001). (G) Table of P5 kidney and body weights, showing decrease in kidney weight without decrease in body weight. (H) Quantification of glomerular number in P40 control and mutant kidneys by acid maceration, showing a significant decrease in glomeruli in mutant kidneys (n=4, p=0.0049). (I) Correlation plot of glomerular number and kidney:body weight ratio, showing a positive correlation. Each n=1 embryo, multiple litters represented per experiment. Bar graphs and violin plot show mean±s.e.m, p values calculated by unpaired two-tailed *t*-test. Scale bars: 50µm (B-C), 20µm (D-E). Correlation was performed using a non-parametric (Spearman) correlation.

Development • Supplementary information

Fig. S3. Early patterning defects in *Ntn1^{SPKO}* are minimized by growth of the kidney, but effects on ramified branching remain.

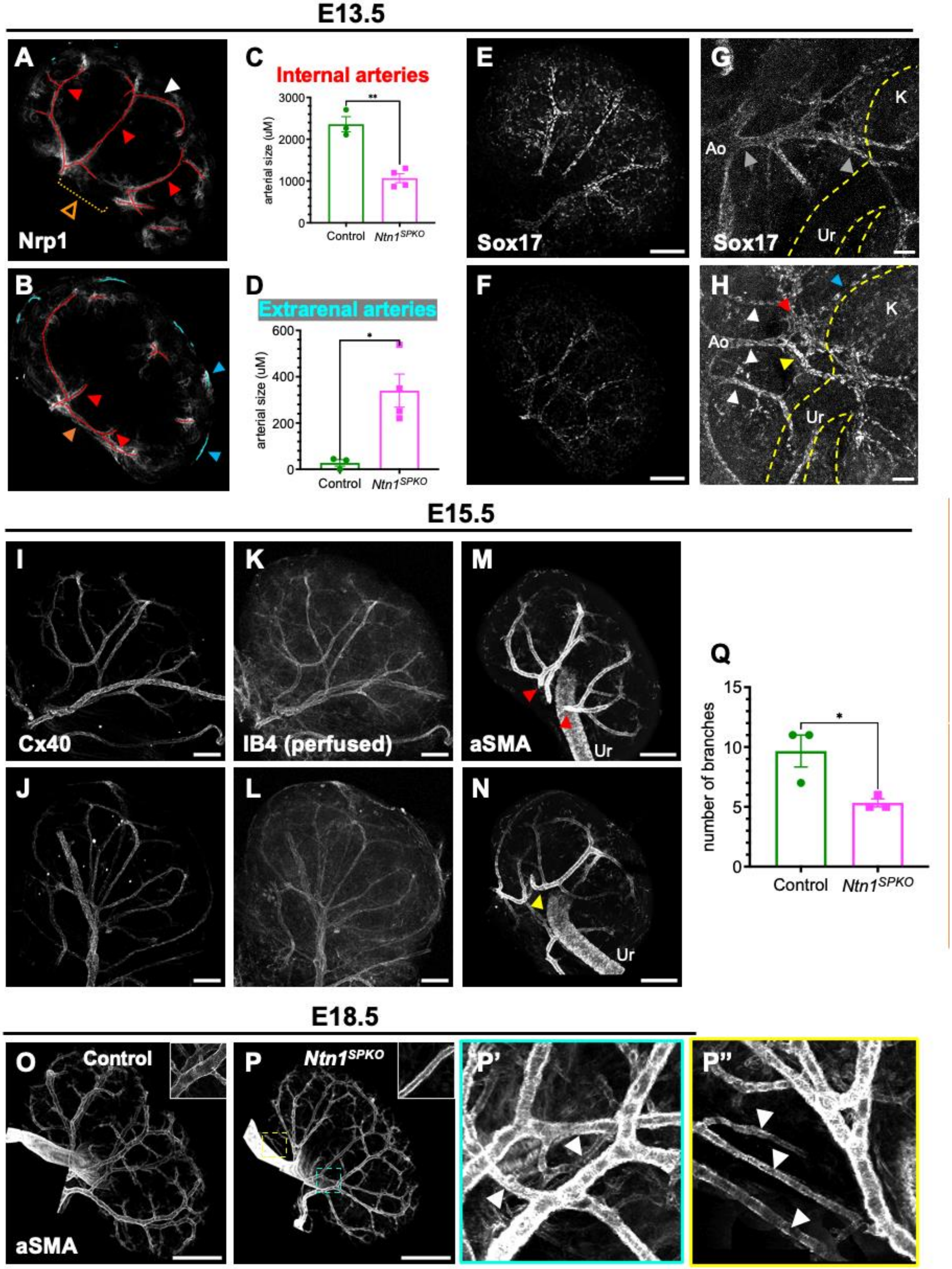

Development • Supplementary information

Fig. S3. Early patterning defects in *Ntn1^SPKO^* are minimized by growth of the kidney, but effects on ramified branching remain. (A-B) Max intensity projection through middle third of the kidney (excluding dorsal and ventral thirds) of Nrp1 WMIF **(Fig.3C,D)** showing decreased internal arteries (red arrowhead, traced in red lines) in $Ntn1^{SPKO}$ kidneys. White arrowhead indicates arcuate artery in control (but not mutant) kidney at the corticomedullary junction. Orange arrowhead indicates an ectopic collateral artery near the hilum of the kidney between intra-renal arteries in $Ntn1^{SPKO}$ kidneys. Orange bracket and unfilled arrowhead indicates lack of collateral arteries in control kidneys. Blue arrowheads and traced arteries indicate extra-renal arteries present only in $Ntn1^{SPKO}$ kidneys. (C) Quantifications of internal arteries in the middle third of control and $Ntn1^{SPKO}$ kidneys, showing decreased arterial length in mutants (n=4, p=0.0012). (D) Quantification of external arteries in control and $Ntn1^{SPKO}$ kidneys, showing increased extrarenal arteries in mutants (n=4, p=0.0147). (E-F) WMIF of E13.5 control and $Ntn1^{SPKO}$ kidneys for Sox17, an arterial marker, showing lower Sox17 levels in mutants. (G-H) WMIF for Sox17 in abdominal (proximal) aortic systems of control and $Ntn1^{SPKO}$ embryos, showing excessive branching of the renal artery before entering the kidney. Grey arrowhead indicates the normal renal artery branching off the aorta. White arrowheads indicate additional aortic branches that connect to the renal artery via additional collaterals (yellow arrowhead). Extra-renal arteries shown with blue arrowhead. Ao=abdominal aorta, K=kidney, Ur=ureter. (I-J) WMIF for Cx40 in E15.5 control and $Ntn1^{SPKO}$ kidneys, showing that most arteries in mutants are $Cx40^{+}$ at this stage. (K-L) WMIF for perfused IB4, showing blood flow into arteries of both control and $Ntn1^{SPKO}$ kidneys. (M-N) WMIF for alpha smooth muscle actin (aSMA) in E15.5 control and $Ntn1^{SPKO}$ kidneys, showing continued arterial mispatterning, but shorter relative distances of arterial entry to the ureter, which also stains for aSMA. (O,P) WMIF for smooth muscle covered arteries in E18.5 control and $Ntn1^{SPKO}$ kidneys, showing normalizing corticomedullary patterning of mature smooth muscle covered arteries, but thinner vessels in mutants (insets). (P',P") Max intensity projections of the hilar region of $Ntn1^{SPKO}$ kidneys, showing rare, but present, collateral arteries that remain (white arrowheads). Fluorescence from ureteric smooth muscle was manually removed using ImageJ before projection. (Q) Quantification of E15.5 arterial branches in control and mutant kidneys, showing decreased branching without Ntn1 (n=3, p=0.0344). Each n=1 embryo, multiple litters represented per experiment. Bar graphs show mean±s.e.m, p values calculated by unpaired two-tailed *t*-test. Scale bars: 50µm (G,H) 100µm (A-B, E-F), 200µm (I-N), 500µm (O-P).

Development • Supplementary information

Fig. S4. *Ntn1^{SPKO}* kidneys exhibit defective vSMC throughout development.

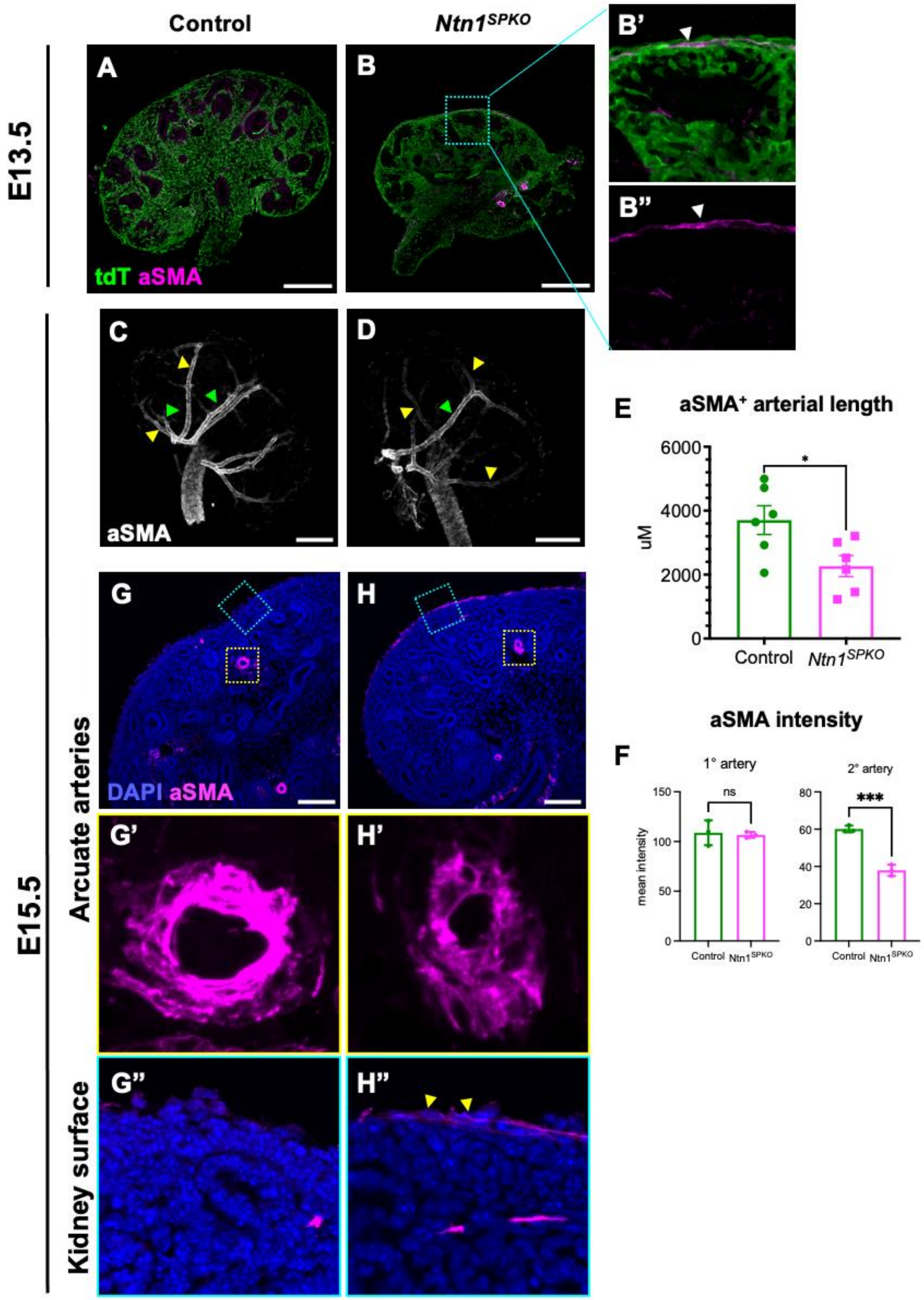

Development • Supplementary information

Fig. S4. *Ntn1^{SPKO}* kidneys exhibit defective vSMCs throughout development. (A-B”) IF staining for aSMA and tdTomato in Cre control and *Ntn1^{SPKO}* kidneys with a Cre-dependent tdTomato reporter marking Foxd1 derivatives, showing tdT expression within ectopic smooth muscle cells at the kidney surface (inset B’,B”, white arrowhead). (C-D) WMIF of E15.5 control and *Ntn1^{SPKO}* kidneys for aSMA, showing coverage of primary arteries with smooth muscle (green arrowheads), but decreased smooth muscle coverage of secondary branches of these arteries (yellow arrowheads). (E) Total length of smooth muscle-covered arterial length measured by aSMA WMIF in kidneys such as (C,D), showing decreased total length of smooth muscle covered arteries in *Ntn1^{SPKO}* kidneys. (F) Quantification of C,D smooth muscle coverage of primary arteries (arteries entering the kidney or equivalent order) and secondary branches (branches of primary arteries), measured by mean intensity of max intensity projection within 25µm (primary) or 10µm (secondary) of arteries (n=3, p=0.7906 (primary) or p=0.0004 (secondary)). (G,H) Immunostaining for aSMA on E15.5 control and *Ntn1^{SPKO}* kidney sections, showing decreased aSMA intensity of arcuate arteries in mutants (G’,H’), and perdurance of ectopic smooth muscle at the kidney surface despite partial restoration of arterial vSMC coverage (G”-H”, yellow arrowheads). Each n=1 embryo, multiple litters represented per experiment. Bar graphs show mean ±s.e.m, p values calculated by unpaired two-tailed *t*-test. Scale bars: 200µm (A-D), 100µm (G-H). Statistical comparison was done using Student’s t-test.

Fig. S5. NG2+ pericyte association with blood vessels is normal at E18.5 in *Ntn1^SPKO^* kidneys.

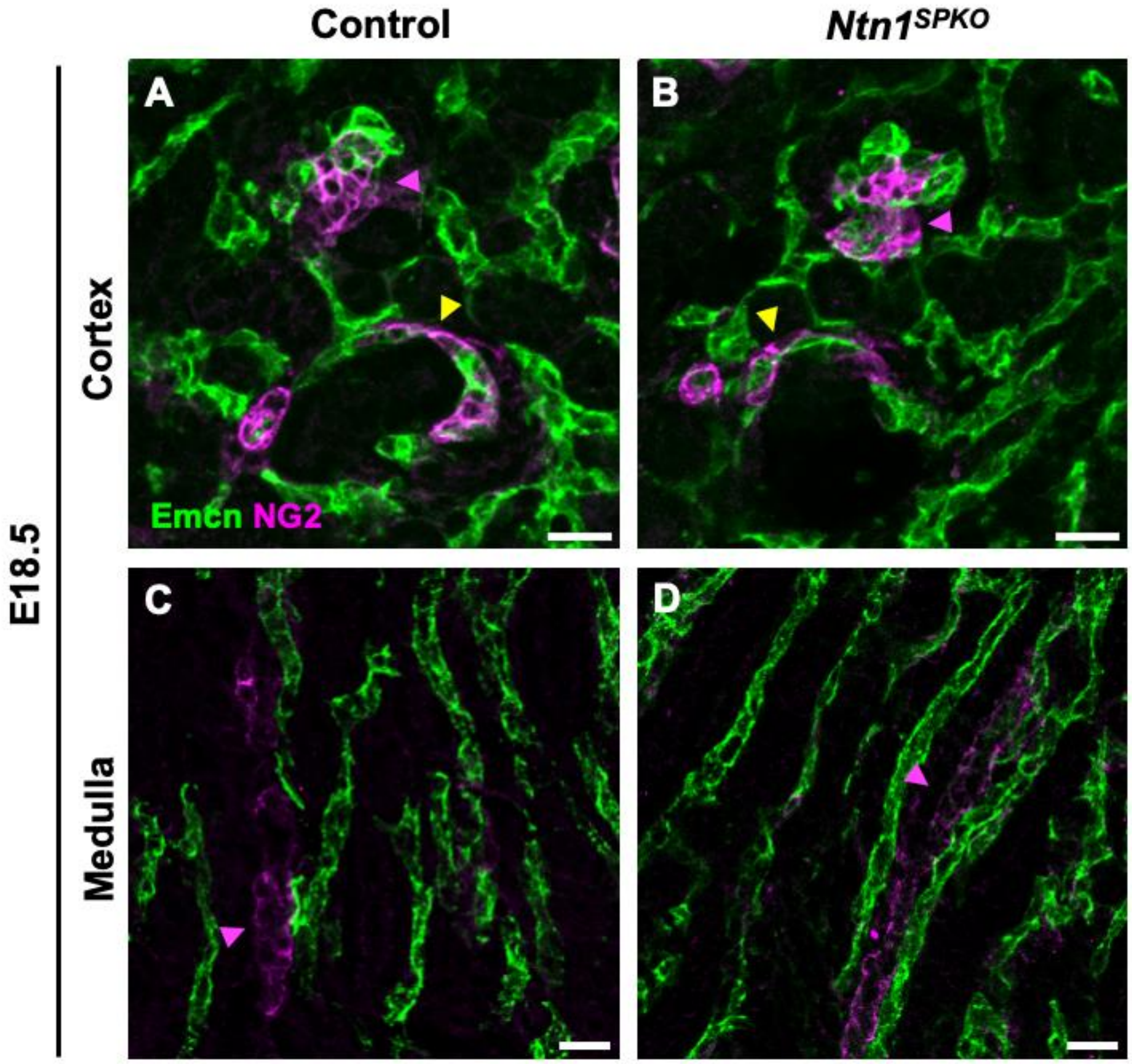

Fig. S5. NG2$^+$ pericyte association with blood vessels is normal at E18.5 in *Ntn1^{SPKO}* kidneys. (A,B) Immunofluorescence of cortex of E18.5 control and mutant kidneys for Emcn and NG2, showing NG2$^+$ pericytes around peritubular capillaries (yellow arrowheads), as well as in glomerular mesangial cells (magenta arrowheads). (C,D) Immunofluorescence of medullary region of E18.5 control and mutant kidneys for Emcn and NG2, showing NG2$^+$ pericytes around descending vasa recta (magenta arrowhead) next to Emcn$^+$ ascending vasa recta. Scale bars: 20µm (A-D).

Fig. S6. Klf4 is expressed in a subset of Foxd1⁺ stromal progenitors throughout nephrogenesis to regulate smooth muscle differentiation.

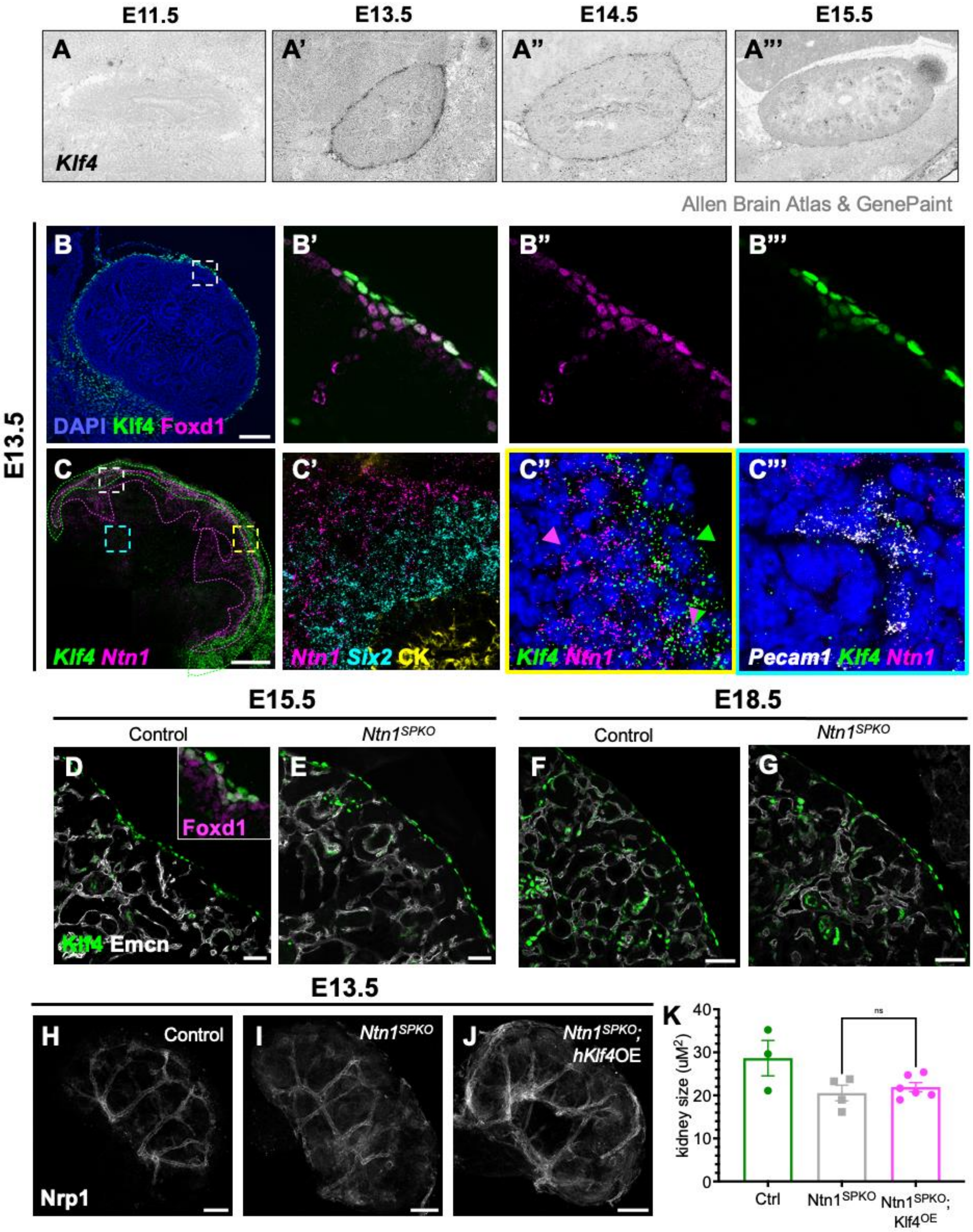

Development • Supplementary information

Fig. S6. Klf4 is expressed in a subset of Foxd1^{+} stromal progenitors throughout nephrogenesis to regulate smooth muscle differentiation. (A) Publicly available *in situ* hybridization of E11.5-E15.5 *Klf4*, showing tight temporal control of expression within cortical stromal cells of the kidney (from Allen Brain Atlas and Genepaint). (B) Immunostaining on control E13.5 kidney sections for Klf4, showing expression in the periphery of the cortex, where the stromal progenitors are localized. (B') Zoom view showing strong colocalization of Klf4 and Foxd1 protein in stromal progenitors nearer the periphery. Single channel views (B",B"') show that stromal progenitors further from the periphery express lower levels of Klf4. (C) RNAscope Hiplex hybridization for *Klf4 and Ntn1* on E13.5 control kidney sections, showing RNA expression in the cortex of the kidney. Dotted lines indicate approximate zones of expression, showing overlapping but distinct patterns of expression, with *Klf4* being solely expressed in the outermost layers of stromal cells and *Ntn1* being expressed further into the kidney. (C') Zoom view of the nephrogenic zone, showing lack of overlap between *Ntn1* and *Six2* expression, as well as lack of *Ntn1* RNA in the cytokeratin^{+} ureteric bud tip. (C") Zoom view of the stromal progenitors, showing cells expressing only *Klf4* at the periphery, only *Ntn1* further from the periphery, and cells expressing both genes in the middle of the stromal progenitors. (C"') Zoom view of a *Pecam1*$^{+}$ blood vessel, showing low levels of *Klf4* expression in endothelial cells at this time point. (D,E) Immunostaining of E15.5 control and *Ntn1*^{SPKO} kidney sections for Klf4, Foxd1 and endomucin, showing fewer stromal progenitors being Klf4^{+} (inset), and a significant amount of Klf4 expression in endothelial cells in both control and mutant kidneys. (F,G) Immunofluorescence of E18.5 control and *Ntn1*^{SPKO} kidney sections for Klf4 and endomucin, showing restriction of stromal Klf4 to the very peripheral layer of cells and strong expression in endothelial cells by this timepoint. (H-J) WMIF of control, *Ntn1*^{SPKO}, and *Ntn1*^{SPKO}; *Klf4*^{OE} kidneys for Nrp1, showing no rescue of arterial patterning with Klf4 overexpression. (K) Quantification of kidney size, showing no rescue of decreased kidney size, with Klf4 overexpression (n=6, p=0.5021). Each n=1 embryo, multiple litters represented per experiment. Bar graphs show mean±s.e.m, p values calculated by unpaired two-tailed *t*-test. Scale bars: 50µm (D-G), 100µm (B,C,H-J)

Fig. S7. ***Klf4^SPKO^*** **does not recapitulate some features of the** ***Ntn1^SPKO^*****, including arterial remodeling and perfusion defects and decreased kidney size.**

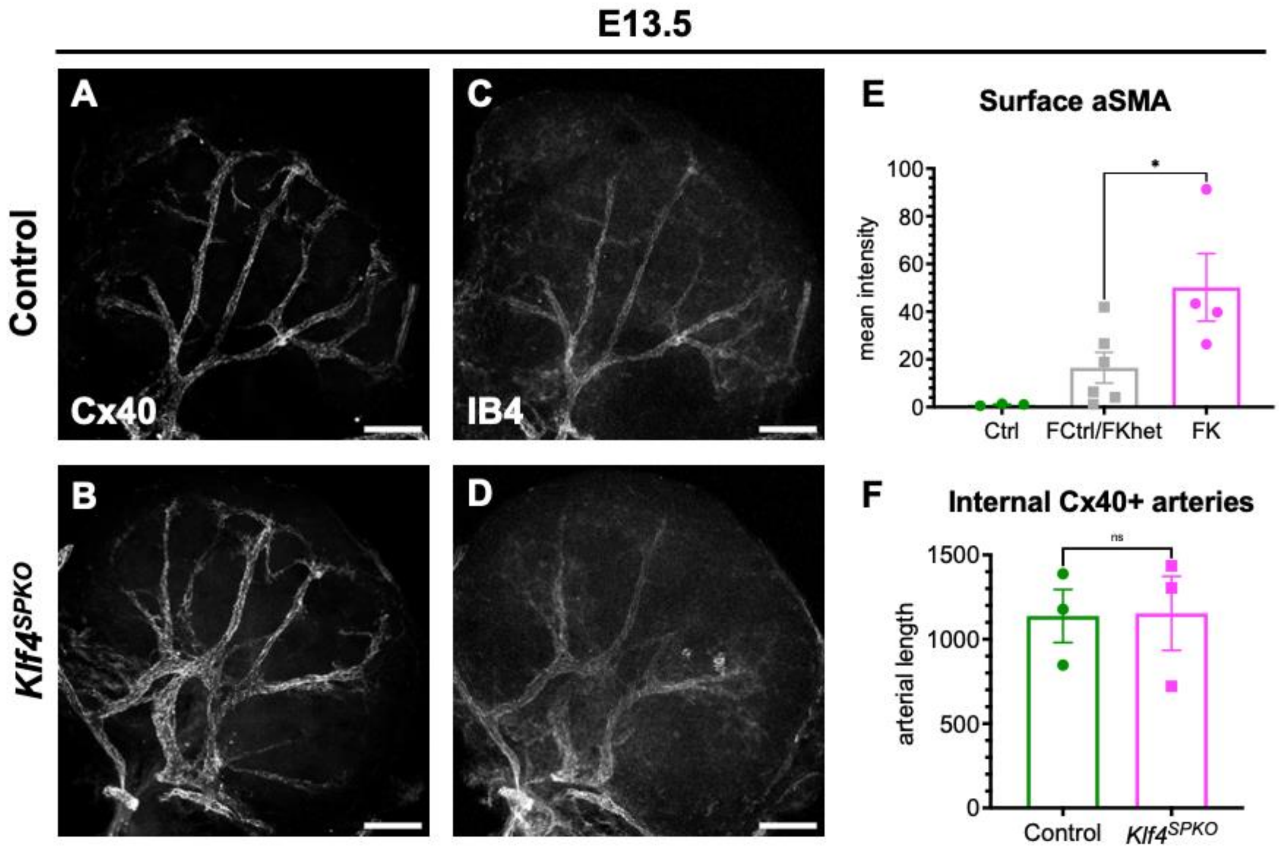

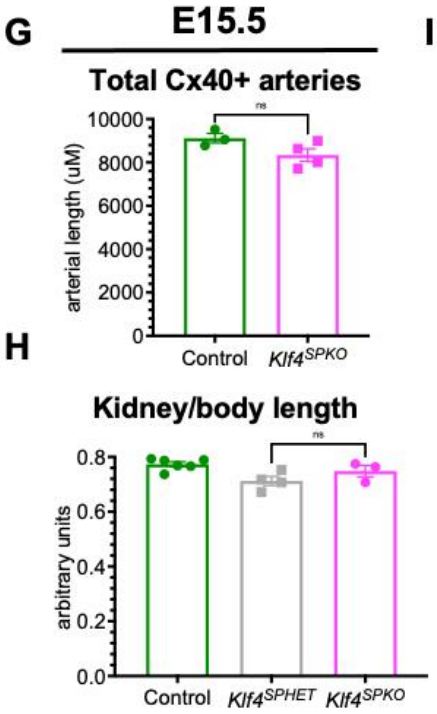

I

	*Ntn1^SPKO^*	*Klf4^SPKO^*	*Ntn1^SPKO^ Klf4OE*
Early renal arterial patterning	Disrupted **(Fig. 3E-G, S3A-J)**	Largely intact **(Fig. S7A,B,F)**	Disrupted **(Fig. S6H-J)**
Perfusion within kidney	Decreased **(Fig. 3C-D)**	Intact **(Fig. S7C,D)**	N/A
Ectopic differentiation of smooth muscle	Present **(Fig. 4A-C)**	Present **(Fig. 7C,D, S7E)**	Absent **(Fig. 6H-K)**
Branching of arterial tree (ramification)	Decreased **(Fig. S4Q)**	Decreased **(Fig. 7G,H,J)**	N/A
Arterial smooth muscle coverage	Decreased **(Fig. 4C, S4E, F)**	Decreased **(Fig. 7I)**	Decreased **(Fig. 6J)**
Kidney size	Decreased **(Fig. 2H,I, S2A)**	Intact **(Fig. S7H)**	Decreased **(Fig. S6K)**

Development • Supplementary information

Fig. S7. Klf4^{SPKO} does not recapitulate some features of the Ntn1^{SPKO}, including arterial remodeling and perfusion defects and decreased kidney size. (A,B) WMIF for Cx40 on E13.5 control and *Klf4^{SPKO}* kidneys, showing no change in Cx40 expression, unlike *Ntn1^{SPKO}* kidneys. (C,D) WMIF for perfused IB4 in 13.5 control and *Klf4^{SPKO}* kidneys, showing no change in perfusion. (E) Quantification of surface smooth muscle coverage in E13.5 control vs *Klf4^{SPKO}* kidneys, measured by mean pixel intensity within 40µm of the surface, showing increased ectopic smooth muscle in mutants (n=4, p=0.0405). (F) Quantification of length of Cx40+ arteries in the middle third of E13.5 control and *Klf4^{SPKO}* kidneys, showing no significant change in internal artery length (n=3, p=0.9552). (G) Quantification of total Cx40+ arterial length in E15.5 control and *Klf4^{SPKO}* kidneys, showing no significant change in overall arterial length (n=4, p=0.1003). (H) Quantification of kidney/body length ratio in control and *Klf4^{SPKO}* kidneys, showing no change in kidney size (n=3, p=0.2298). (I) Table listing phenotypes observed in *Ntn1^{SPKO}, Klf4^{SPKO}, Ntn1^{SPKO}; Klf4^{OE}* kidneys, along with associated figures. Each n=1 embryo, multiple litters represented per experiment. Bar graphs show mean±s.e.m, p values calculated by unpaired two-tailed *t*-test. Scale bars: 100µm (A-D).

Table S1. List of antibodies used in this study.

Primary antibodies

Target	Company	Catalog no.	Dilution (IF and WMIF)å	Antigen Retrieval (for paraffin IF)
Ntn1	abcam	ab126729	1:100, 1:1000 (WB)	Buffer A, Tris-EDTA
β-actin	Cell Signaling Technologies	3700	1:2000 (WB)	N/A
Six2	Proteintech	11562-1-AP	1:100	Buffer A
Meis1/2/3	Active Motif	39795	1:100	Buffer A
Calbindin	Sigma	C9848	1:100	N/A
DBA	Vector	B-1035-5	1:100	N/A
Pax8	Cell Signaling Technologies	9857	1:100	Buffer A
NCAM	Sigma	C9672	1:100	N/A
Cx40	Alpha Diagnostics	CX40-A	1:100	N/A
Cx40	Invitrogen	36-500	1:100	N/A
Nrp1	R&D Systems	AF566	1:100	N/A
Emcn	Santa Cruz	sc-65495	1:100	N/A
Sox17	R&D Systems	AF1924	1:100	Buffer A
Isolectin B4-Alexa Fluor 488	Invitrogen	I21411	1:50	N/A
aSMA-Cy3	Sigma	C6198	1:200	Buffer A,B
Cnn1	Abcam	ab46794	1:100	N/A
tdTomato	MyBioSource	MBS448092	1:100	N/A
NG2	Sigma	AB5320	1:100	Buffer A,B
Klf4	R&D Systems	AF358	1:100	Buffer A,B
Klf4	abcam	ab214666	1:100	Buffer A
Foxd1	LSBio	LS-B6453	1:100	Buffer A

Secondary antibodies (Concentrations: 1:500 for IF, WMIF, 1:10000 for WB)

Host	Target	Conjugate	Company	Catalog
Donkey	Goat	Alexa Fluor™ 555	Invitrogen	A11055
Donkey	Goat	Alexa Fluor Plus™ 647	Invitrogen	A32849
Donkey	Rabbit	Alexa Fluor™ 488	Invitrogen	A21206
Donkey	Rabbit	Alexa Fluor™ 555	Invitrogen	A31572
Donkey	Rabbit	Alexa Fluor™ 647	Invitrogen	A31573
Donkey	Rat	Alexa Fluor Plus™ 647	Invitrogen	A48272
Donkey	Rat	Alexa Fluor™ 488	Invitrogen	A21208
Streptavidin	Biotin	Alexa Fluor™ 488	Invitrogen	S32354
Donkey	Mouse	Alexa Fluor™ 488	Invitrogen	A21202
Donkey	Mouse	Alexa Fluor™ 647	Invitrogen	A31571
Donkey	Mouse	HRP	Santa Cruz	SC-2318
Donkey	Rabbit	HRP	Jackson Immuno	711-035-152
