# Supplementary material for: Stromal netrin 1 coordinates renal arteriogenesis and mural cell differentiation
Source: Development. 2023 Nov 24;150(22):dev201884. doi: 10.1242/dev.201884 (PMC10690105; doi:10.1242/dev.201884)
Supplement: Supplementary file 1 [file develop-150-201884_review_history.pdf]

Stromal netrin-1 coordinates renal arteriogenesis and mural cell differentiation

Peter M. Luo, Xiaowu Gu, Christopher Chaney, Thomas Carroll and Ondine Cleaver
DOI: 10.1242/dev.201884

Editor: Liz Robertson

Review timeline
Original submission: 14 April 2023
Editorial decision: 1 June 2023
First revision received: 5 September 2023
Accepted: 2 October 2023

Original submission

First decision letter

MS ID#: DEVELOP/2023/201884

MS TITLE: Stromal netrin-1 coordinates renal arteriogenesis and mural cell differentiation

AUTHORS: Peter M Luo, Xiaowu Gu, Christopher Chaney, Thomas J. Carroll, and Ondine Cleaver

I have now received all the referees' reports on the above manuscript, and have reached a decision. The referees' comments are appended below, or you can access them online: please go to BenchPress and click on the 'Manuscripts with Decisions' queue in the Author Area.

As you will see, the referees express considerable interest in your work, but have some significant criticisms and recommend a substantial revision of your manuscript before we can consider publication. If you are able to revise the manuscript along the lines suggested, which may involve further experiments, I will be happy receive a revised version of the manuscript. Your revised paper will be re-reviewed by one or more of the original referees, and acceptance of your manuscript will depend on your addressing satisfactorily the reviewers' major concerns. Please also note that Development will normally permit only one round of major revision. If it would be helpful, you are welcome to contact us to discuss your revision in greater detail. Please send us a point-by-point response indicating your plans for addressing the referee’s comments, and we will look over this and provide further guidance.

As your paper was submitted alongside the one from the O'Brien lab, the Reviewers have indicated that they think it would be appropriate if your laboratories could discuss and share the data contained in your respective papers and address any discrepancies in interpretation. Lori's paper has also been returned for revision to address the concerns raised by the Reviewers. You might also want to consult with each other to ensure that the revised versions of your papers are re-submitted at the same time.

Please attend to all of the reviewers' comments and ensure that you clearly highlight all changes made in the revised manuscript. Please avoid using 'Tracked changes' in Word files as these are lost in PDF conversion. I should be grateful if you would also provide a point-by-point response detailing how you have dealt with the points raised by the reviewers in the 'Response to Reviewers' box. If you do not agree with any of their criticisms or suggestions please explain clearly why this is so.

© 2023. Published by The Company of Biologists under the terms of the Creative Commons Attribution License (https://creativecommons.org/licenses/by/4.0/).

Reviewer 1

Advance summary and potential significance to field

This manuscript by Luo et al interrogates the role of stroll Netrin-1 in renal arteriogenesis and mural cell differentiation. The authors use a combination of molecular and genetic techniques to define the role of this molecule in kidney development. Much of the field of kidney development focuses on the role of the various kidney progenitors with few focusing on the kidney developing vasculature. This field aptly describes normal vascular formation with sophisticated imaging techniques and further elaborates the cellular interactions that take place between the endothelium and smooth muscle that so closely associates.

Comments for the author

The authors have described a new signaling phenomenon that exists between the stromal progenitor cells and the developing vasculature that drives maturation and patterning of this critical cell compartment. Overall this manuscript is well written and concise. It is technically sound and mechanistically developed. However there are some key questions that need to be addressed. These are listed below.

1. There is a distinct expression pattern that exists with Netrin-1 in terms of the protein and the RNA. There appear to be vesicular like structures that exist from the protein analysis that converge from the stroma through to the UB. This needs to be further elucidated and discussed and put into context. Do you think that there is non-cell autonomous signaling between one compartment and another where the message is made in one compartment and transcribed in another? Potentially live cell imaging could show the transport of extracellular vesicles to further define.

2. The authors do a nice job of phenotypically describing the mutants and the kidneys are convincingly smaller and the kidney/body weight ratio is less. However seeing the raw numbers for the body weight would be helpful as it is not clear whether there is an overall smaller embryo or is this truly a delay in nephrogenesis, this could be tested to see if the cessation of nephrogenesis is altered. Could you also measure kidney and body length and width that would add strength to this analysis.

3. In figure 3 the data related to the different types of vessels that are present including intra, extra and penetrating is compelling. Would there be a way to quantitate this to determine the prevalence and difference compared to the control?

4. IF shows clear changes in the abundance of aSMA in the developing kidneys and surrounding the developing vasculature. However, IHC may show more pronounced changes and allow one to appreciate precise locations and also coupling this with an expanded look at different developing time points would seem appropriate.

5. It is intriguing that the pericyte phenotype normalizes by E18.5, why do the authors think this is the case and does this decrease the importance of this phenotype?

6. The data with KLF4 although intriguing seems to be over emphasized and under developed. With the overexpressor there is a partial rescue but a limited interrogation of vascular remodeling and no assessment in changes to kidney size or nephron number was performed. It begs the question of alternative mechanisms and this should be explored.

7. This group has focused extensively on the various stromal derivatives although with this manuscript they have lumped all the distinct derivatives into a single Foxd1 positive package. A more in depth discussion of the derivatives and potential interface with Netrin-1 signaling is required.

8. To really delve into the perceived role of Netrin-1 in driving differentiation and arteriogenesis an in vitro model showing how the knockout cells interface with endothelium and angiogenesis would be informative.

© 2023. Published by The Company of Biologists under the terms of the Creative Commons Attribution License (https://creativecommons.org/licenses/by/4.0/).

Reviewer 2

Advance summary and potential significance to field

Netrin-1 secreted by the renal stroml population plays a role in patterning the renal vascular bed at early, but not late stages of development. Since the patterming defects are transitory and vascular bed mormalized as the kidney begins to function, the study does not really add much to the literature as submitted.

Comments for the author

The generation of functional cell-derived tissues is dependent on an understanding of the molecular mechanism controlling organ specific vascular patterning. The ms. submitted by Luo et al investigates the role of the secreted signaling factor, Netrin1, in this process as the kidney develops.

A cursory analysis of renal epithelial development indicates that an absence of netrin 1 signaling results in small kidney size despite prolonged nephrogenesis. The primary focus of the study is a more thorough analysis of the renal vascular network in the absence of netrin 1 signaling. This part of the study demonstrates that netrin1 plays a role in vascular patterning at early stages of development, and that Klf4 is downstream of netrin signaling. However, these results are not so interesting as arterial vascular patterning at later stages of development appears to normalize. Due to the complexity of the mature renal vascular bed, a thorough analysis of the normalized arterial tree in the mature mouse is difficult. That the netrin-1 mice live into adulthood suggests that the netrin-1 null vascular network is sufficient for renal function. Blood flow studies at early and late stages of development would greatly strengthen the hypothesis that the vascular network is defective at early, but not late stages of development. Notably, as stated in the discussion these experiments have been performed but are not included in ms.

In summary, this ms. would be of great interest if it investigated the mechanisms guiding the normalization of arterial patterning defects observed at early stages of development. As it stands now, it is focused solely on a transient phenotype. Moreover, many of the experiments are done in a superficial manner and interpretation of some crucial results flawed.

Specific comments:

- ***“Loss of renal netrin-1 impairs recruitment of arterial smooth muscle and premature differentiation at the surface of the kidney”*** More definitive smooth muscle markers such as myh11 or calponin are required to support the hypotheses that that netrin1 ablation results in ectopic localization and precocious smooth muscle differentiation.
- Although Connexin40 expression seems to be decreased in mutants at 13.5, differential expression at later stages of development is not reported.
- The Klf4 rescue experiments are very superficial. Is kidney size also restored?

Reviewer 3

Advance summary and potential significance to field

Luo et al. investigate the molecular mechanisms that direct vascular patterning of the developing kidney. They focus on the guidance molecule Netrin1, and generated and analyzed mice harboring a stromal-progenitor Netrin1 KO. They do a very thorough job of describing the multiple defects resulting from this deletion, that affect kidney size, epithelial branching and nephrogenesis, as well as angiogenesis and arteriogenesis. They find that multiple renal cell types are affected by loss of stromal Netrin1, including epithelial cells, endothelial cells, pericytes and vascular smooth muscle cells. Bulk RNA sequencing identified KLF4 downregulation in Ntn1 kos, and a stromal progenitor KLF4 knockout phenocopies the Ntn1 ko. Further, conditional KLF4 overexpression rescues smooth

© 2023. Published by The Company of Biologists under the terms of the Creative Commons Attribution License (https://creativecommons.org/licenses/by/4.0/).

muscle cell differentiation in Ntn1 mutants. The authors propose a novel stromal-derived Ntn1-KLF4 axis that regulates proper nephrogenesis and vascularization of the embryonic kidney.
The work is well written and illustrated, and reveals for the first time a vascular phenotype in Ntn1 mutant kidneys. This phenotype is complex, therefore mechanisms of action might be difficult to determine, as exemplified in the accompanying paper of Honeycutt et al. Nevertheless, assembly of organ-specific vasculature remains poorly understood, and this work is important as it reveals novel roles of Netrin1 in renal vasculature development.

Comments for the author

Before publication can be recommended, the following issues should be addressed.

1- Vascular phenotypes in stromal Ntn1 mutants are shown to be present at E13.5 but are resolved at E18.5 in this manuscript. Honeycutt et al observe persistent defects at postnatal stages, please clarify. Likewise, please provide quantifications for vascular branchpoints and coverage at different time points. Honeycutt Fig.4 shows similar values for vasculature between control and mutant at E15.5, which is at odds with the severe arterial branching defects shown in this manuscript.

2- The mechanism by which Ntn1 regulates Klf4 remains rather elusive and should be improved. Klf4 was shown to regulate Netrin-1 promoter activity in other cell types (doi: 10.1038/ncomms8398). The authors claim that KLF4 is downstream of Ntn1, but they should also check Ntn1 levels in Klf4SPKO and cre- kidneys.

4- Are KLF4 levels normalized over time in Ntn1SPKO kidneys, along with their phenotype? Similarly, is the phenotype seen in KLF4SPKO transient like the one seen in NTN1SPKO kidneys?

5- The authors state that both Ntn1SPKO and KLF4SPKO kidneys exhibited penetrating arteries that looped and connect to intrarenal arteries in some cases, presence of extrarenal arteries and ectopic SMA coverage. It would be useful to quantify these phenotypes in both NTN1SPKO and KFL4SPKO to understand to which extent these genetic deletions phenocopy each other.

6- The authors claim that the suppression of branching is due to the excess contractile SMC at the developing kidney periphery. Does NTN1;KLF4 OE rescue arterial branching in addition to the rescue of Ntn1SPKO induced aSMA coverage at the kidney periphery? Overall, a more detailed characterization of NTN1;KLF4 OE kidneys is needed to understand the extent of KLF4 involvement in NTN1-induced vascular patterning of the developing kidney.

7- Supp Fig S3 G,H and S4 C,D are the same, why? please replace one set of images.
Fig.3C and S3C also show same image, please replace one, and provide a table of the number of kidneys analyzed in this work.

First revision

Author response to reviewers' comments

Point-by-point response to reviewers
We thank the reviewers for the genuinely helpful comments. We are grateful for the suggestions, as we feel addressing them has improved our manuscript significantly. We have carried out many new experiments, have added many new panels within revised figures and one new supplemental figure, and have extensively addressed reviewers' points in entirely new writing throughout the manuscript. Below is a point-by-point response to the two reviewers' concerns. Our responses are shown in black below (reviewer comments in blue), and changes to the manuscript in blue text. We hope reviewers will now find the revisions acceptable and the work now worthy of publication in *Development*.

Response to Reviewers:

© 2023. Published by The Company of Biologists under the terms of the Creative Commons Attribution License (https://creativecommons.org/licenses/by/4.0/).

Reviewer 1 Advance Summary and Potential Significance to Field:
This manuscript by Luo et al interrogates the role of stroll Netrin-1 in renal arteriogenesis and mural cell differentiation. The authors use a combination of molecular and genetic techniques to define the role of this molecule in kidney development. Much of the field of kidney development focuses on the role of the various kidney progenitors with few focusing on the kidney developing vasculature. This field aptly describes normal vascular formation with sophisticated imaging techniques and further elaborates the cellular interactions that take place between the endothelium and smooth muscle that so closely associates.

Reviewer 1 Comments for the Author:
The authors have described a new signaling phenomenon that exists between the stromal progenitor cells and the developing vasculature that drives maturation and patterning of this critical cell compartment.
Overall this manuscript is well written and concise. It is technically sound and mechanistically developed. However, there are some key questions that need to be addressed. These are listed below.

1. There is a distinct expression pattern that exists with Netrin-1 in terms of the protein and the RNA. There appear to be vesicular like structures that exist from the protein analysis that converge from the stroma through to the UB. This needs to be further elucidated and discussed and put into context. Do you think that there is non-cell autonomous signaling between one compartment and another where the message is made in one compartment and transcribed in another? Potentially live cell imaging could show the transport of extracellular vesicles to further define.

We agree with the reviewer that this is really interesting! We have also long noticed that immunostaining for Ntn1 show it as punctae, and that some of it clusters around the UB. As we lay out in the manuscript, however, we believe netrin-1 mRNA is solely made in the stroma because our *in situ* hybridization data shows a lack of netrin-1 mRNA outside of the stromal cells. As netrin-1 is a secreted protein, we interpret the punctate immunofluorescent (IF) pattern as representing netrin-1 protein secreted into the ECM of the cortical stroma, where it is free to diffuse. (Our images are maximum intensity projection and the protein is primarily along the surface of the UB). We speculate that the enrichment of netrin-1 protein on the UB may reflect either as a signaling event to the UB (as the reviewer suggests) or that is a sequestration event to prevent further protein diffusion within the stroma. However, at this juncture, it is an open question.

We appreciate the possibility brought up by the reviewer that this pattern could be interpreted as Ntn1 being transcribed in the stroma and mRNA transported to the UB via exosomes or extracellular vesicles. This is possible, however would be technically difficult to parse out. Live imaging experiments to determine where Ntn1 protein is made would require a Ntn1-GFP fusion construct being stably integrated (via transgenesis), for example. In addition, we do not see mRNA (that might be transferred to the epithelial cells) within the epithelial compartment by either *in situ* hybridization or RNAscope analysis. We posit instead that secreted Ntn1 protein is being taken up by the UB epithelium. Please see added discussion on this on page 11. While interesting, we respectfully feel this is thus outside the scope of our study and live imaging with the appropriate tools would extend beyond the timeframe allowed for revisions on this manuscript.

Page 11: *"Ntn1 protein, by contrast, is found diffusely surrounding both stromal and epithelial cells in the nephrogenic zone. The presence of protein in and around cells without transcripts shows that Ntn1 is secreted from SPs into the cortex. In particular, Ntn1 protein appears enriched at the terminal UB tips. It is possible that the UB serves as a ligand sink, constraining Ntn1 to the cortical nephrogenic zone throughout development. However, given the decrease in UB branching in Ntn1^{SPKO} kidneys, a direct signaling role cannot be excluded."*

2. The authors do a nice job of phenotypically describing the mutants and the kidneys are convincingly smaller and the kidney/body weight ratio is less. However seeing the raw numbers for the body weight would be helpful as it is not clear whether there is an overall smaller embryo or is this truly a delay in nephrogenesis, this could be tested to see if the cessation of nephrogenesis is altered. Could you also measure kidney and body length and width that would add strength to this

© 2023. Published by The Company of Biologists under the terms of the Creative Commons Attribution License (https://creativecommons.org/licenses/by/4.0/).

analysis.

This is a good point. We recognize that kidney to body weight ratio does not completely preclude the possibility that the overall embryo is delayed. However, weighing embryos is difficult due to variability in water weight (water around and within the embryos at dissection), and drying the embryos would make dissection and kidney retrieval difficult. To circumvent this issue, we have collected new data for embryonic stages to show embryo and kidney length, as requested (please see **Figure S2A**). We have also added data for whole body weights at postnatal day 5 along with kidney weights as requested, showing that there is no significant difference in embryonic development at these embryonic and perinatal stages (please see **Figure S2G**). We discuss these new data on page 4 and 5. We note that Honeycutt et al do length comparisons of kidney sizes at later postnatal stages (Honeycutt et al new Figure 1) and we have aimed not to try to reproduce all their data, in an effort to keep the studies distinct and complementary. Overall, there is a clear robustness of the size phenotype between our models across developmental stages.

Page 4: *"Grossly, Ntn1^{SPKO} kidneys were smaller than those of littermate control embryos **(Fig. 2E,F)**. Analysis of kidney and embryo lengths at E13.5 showed that decreases in kidney size was independent of potential overall embryonic growth delays **(Fig. S2A)**."*

-and-

Page 5: *"Mutant kidneys continued to be smaller after birth, both by visual comparison at P5 **(Fig. 2H, S2G)** and by kidney/body weight ratios **(Fig. 2I, S2G)**."*

3. In figure 3 the data related to the different types of vessels that are present including intra, extra and penetrating is compelling. Would there be a way to quantitate this to determine the prevalence and difference compared to the control?

We thank the reviewer for agreeing that the abnormal vasculature in the *Ntn1^{SPKO}* is striking. We have added quantifications to show differences in penetrating artery anatomy in the form of average distance from the hilum to the entry point/total corticomedullary distance (please see **Figure 3G**).

Intrarenal arteries were difficult to identify and define in all mutants, so we have now instead quantified total length within the interior of the kidney (defined as the middle 1/3 of the kidney, measured width-wise) (please see **Figure S3C**). We also now quantified external arteries by measuring the arterial length at the surface in the middle third of the kidney (please see **Fig. S3D**). Discussion on these new data is found on pages 5 and 6.

Page 5: *"Arteries in Ntn1^{SPKO} kidneys that did enter via the hilum ('intrarenal' arteries) did not progress normally into the kidney **(Fig 3E,F**, red arrowheads**)**. Max intensity projections of the center third of Nrp1 WMIFs showed significantly shorter intrarenal arterial length and excess extrarenal arteries in Ntn1^{SPKO} kidneys **(Fig. S3A-D)**."*

-and-

Page 6: *"We measured the average distance from each arterial entry point to the ureter and divided it by the total corticomedullary distance. At E13.5, this ratio was significantly increased in mutants, but by 15.5 it became insignificant, suggesting that growth of the kidney progressively minimizes the effects of early arterial mispatterning, explaining the restoration of Cx40 and IB4 positivity **(Fig. 3G)**."*

4. IF shows clear changes in the abundance of aSMA in the developing kidneys and surrounding the developing vasculature. However, IHC may show more pronounced changes and allow one to appreciate precise locations and also coupling this with an expanded look at different developing time points would seem appropriate.

We thank the reviewer for the suggestion. We have felt that IF was better than IHC, in that it allows use of multiple antibodies (conjugated to different fluorophores) for co-localization of markers, and we had wanted to show smooth muscle cells in association with blood vessels. However, we have sought to address the reviewer's point using multiplex immunofluorescence staining on sections, which is more sensitive than either whole mount IF or IHC. This has allowed us to visualize localization more precisely and to glean insights into cellular lineage of ectopic smooth muscle cells. We show now that aSMA^{+} cells are indeed at the very periphery of the kidney at E13.5, as assessed by co-staining with nuclear DAPI. By performing co-stains

© 2023. Published by The Company of Biologists under the terms of the Creative Commons Attribution License (https://creativecommons.org/licenses/by/4.0/).

for tdTomato, we show that ectopic smooth muscle cells are stromal cells derived from the Foxd1^{+} stromal progenitors (please see **Fig S4A-B"**). We show that aSMA^{+} cells continue to be localized at the periphery at E15.5 (please see **Fig. S4G-H"**). We also discuss these new stains and the potential identity of these cells on page 7.

Page 7: *"To confirm that ectopic SMCs are derived from the same progenitors that contribute to normal vSMCs, we crossed in a fluorescent reporter allele (LSL-tdTomato) to trace the Foxd1^{GC} lineage in mutants and Cre^{+} controls. At E13.5, immunostaining for tdTomato showed that ectopic SMCs and normal arterial vSMCs (in controls) were indeed derived from Foxd1-expressing SPs **(Fig. S4A-B")**."*

-and-

Also page 7: *"second order vessels (branches off first order branches, including arcuate arteries, yellow arrowheads) showed significantly lower staining intensity in Ntn1^{SPKO} **(Fig. S4C,D**, quantification in **Fig. S4F, and Fig. S4G-H')**. We noted that when present, penetrating arteries had stronger aSMA staining that decreased as entered the kidney. Intriguingly, ectopic aSMA^{+} cells remained present at the kidney surface at E15.5 **(Fig. S4G",H"** yellow arrowheads**)**, and did not resolve to contribute to vascular smooth muscle."*

5. It is intriguing that the pericyte phenotype normalizes by E18.5, why do the authors think this is the case and does this decrease the importance of this phenotype?

We appreciate the question, and we also thought it is intriguing that there is not much difference when looking at NG2^{+} pericytes at E18.5. We think this is because NG2 does not mark the same cells at E18.5 as at E13.5 (when loss of Ntn1 influences NG2 localization). Our own transcriptomic analysis shows that NG2 only marks a subset of pericytes and its expression changes over time (England et al., 2020). To support this observation, we show here (shown here for reviewer, Rev Fig. 1) that expression of NG2 at later time points, such as P6, is relatively low around cortical capillaries, while these were initially surrounded by NG2^{+} pericytes at E18.5. In addition, at E13.5 we show that NG2^{+} cells are not associated with capillary vasculature (please see **Fig. 5A,B)**.

In sum, we do not believe that NG2 is a permanent or specific cell marker for pericytes in the kidney and very likely mark different populations at E13.5 and at E18.5. Thus, the absence of a phenotype when looking at NG2 at E18.5 does not decrease the importance of the phenotype observed at E13.5, but rather stresses the temporal role of Ntn1 in early nephrogenesis. It also underscores the resilience of the stroma to genetic disruptions. To assess how the phenotype develops through time, we would need a lineage tracing method such as the inducible NG2-CreERT2, but such analysis lies outside of the scope and possibility of this study. We have adjusted the discussion of pericytes in the text to clarify (please see pages 8 and 15).

Page 8: *"Surprisingly, NG2 staining was not present around cortical capillaries in either control or Ntn1^{SPKO} kidneys **(Fig. 5A-B')**. Instead, we found that NG2^{+} cells invested around arteries in both control and mutant kidneys (**Fig. 5C-D'**). Costaining for aSMA showed that inner layers of SMCs expressed NG2 (**Fig 5C'**, white arrowhead), but outer layers of mural cells expressed only NG2, with little to no aSMA **(Fig. 5C'**, green arrowhead**)**. NG2^{+} mural cells were still able to invest arteries in Ntn1^{SPKO} kidneys but appeared markedly reduced **(Fig. 5D'**, red arrowheads**)**."*

-and-

Page 13: *"Intriguingly, we find NG2, a marker of pericytes, expressed uniquely in some cells surrounding vSMCs in the periarterial niche. Whether these cells are related to pericytes found later in development around the capillary vasculature is an open question. As vSMCs also express NG2, it is possible that these simply represent immature smooth muscle, but analysis of kidneys lacking Ntn1 demonstrates investment of NG2+ cells without aSMA."*

© 2023. Published by The Company of Biologists under the terms of the Creative Commons Attribution License (https://creativecommons.org/licenses/by/4.0/).

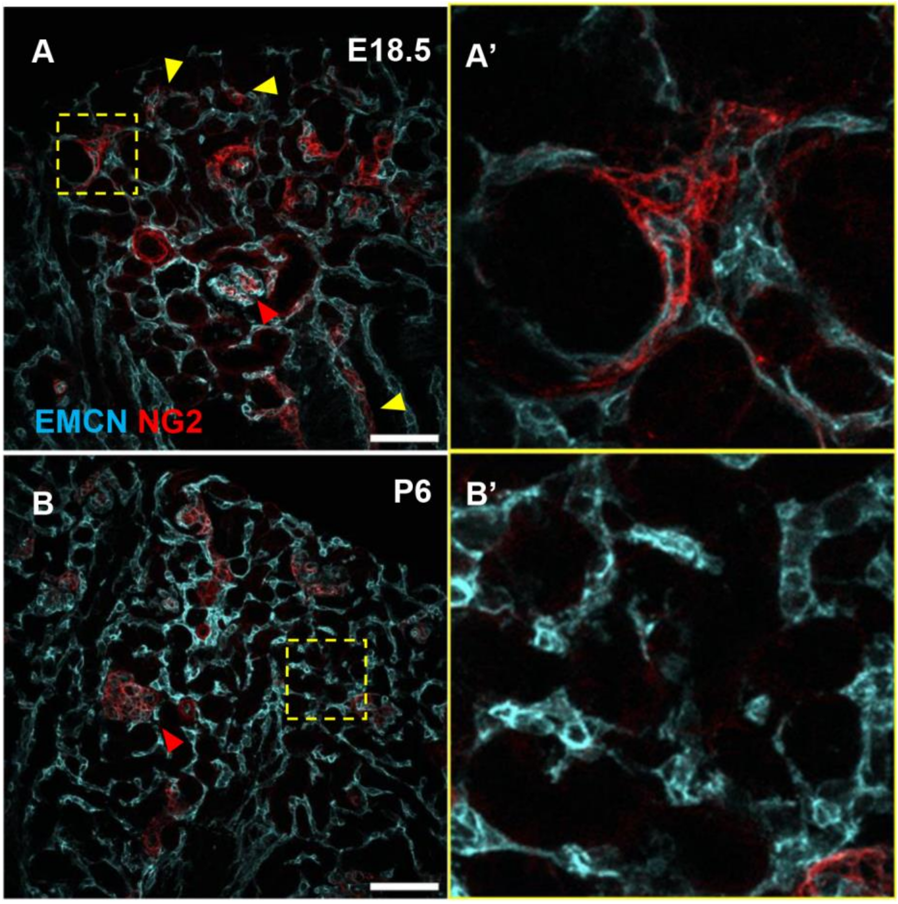

Rev. Fig. 1: Differential expression of NG2 around capillaries at E18.5 and P6. Immunofluorescence for endomucin and NG2 on E18.5 (A) and P6 (B) control kidney sections, showing decreased capillary coverage with NG2+ cells by P6. Yellow arrowheads indicate NG2 coverage of capillaries at E18.5. Red arrowheads indicate glomerular mesangial cells, which are NG2+ at both stages. NG2 not associated with endomucin at either stage is arterial smooth muscle, as arterial endothelial cells do not stain for endomucin.

6. The data with KLF4 although intriguing seems to be over emphasized and under developed. With the overexpressor there is a partial rescue but a limited interrogation of vascular remodeling and no assessment in changes to kidney size or nephron number was performed. It begs the question of alternative mechanisms and this should be explored.

We thank the reviewer for this comment and agree that we had limited our discussion of the Klf4 rescue in our initial manuscript. To expand upon these findings, we have now added data to show that while Klf4 overexpression rescues the expansion of vSMCs at the kidney periphery, it does not rescue kidney size or arterial patterning as assessed by kidney volume and NRP1 staining (please see **Fig S6H-K**). This supports our model that Klf4 regulates smooth muscle differentiation (over the course of kidney arteriogenesis), and that other phenotypes we describe (gross remodeling of the proximal primary renal artery) are likely due to non-Klf4 mediated effects downstream of loss of Ntn1. We have discussed the limitations of this overexpression model, and thus what we can analyze, both in our response to reviewer 3 point 3 (below) and on page 9 and 12. Here, we respectfully argue our study focuses on the role of Ntn1 in limiting mural cell recruitment during renal arteriogenesis. We believe that we have convincingly shown that netrin-1 is required for Klf4 expression within smooth muscle progenitors to allow for proper recruitment to the arteries, and along with Honeycutt, et al, we show that smooth muscle and mural cell recruitment likely promotes branching of the kidney arterial tree (or support/maintain it) throughout nephrogenesis.

© 2023. Published by The Company of Biologists under the terms of the Creative Commons Attribution License (https://creativecommons.org/licenses/by/4.0/).

Page 9: *"Klf4 overexpression did not, however, restore smooth muscle coverage at the arteries, likely due to an inability of vSMC progenitors to differentiate due to Klf4 overexpression. Klf4 overexpression also did not rescue gross arterial patterning defects* ***(Fig. S6H-J)****, nor did it restore kidney size in the Ntn1^{SPKO} background* ***(Fig. S6K)****, suggesting these effects of Ntn1 loss were independent of Klf4."*

-and-

Page 12: *"Notably, Klf4 overexpression does not restore arterial smooth muscle coverage at E13.5. We suspect that the level of overexpression achieved was enough to reduce ectopic differentiation at the surface, but likely too much to then allow proper downregulation of Klf4 at the arteries and differentiation to vSMCs. Alternatively, as the overexpression was performed in a Ntn1^{SPKO} background, other Klf4- independent effects of Ntn1 ablation could result in a lack of signals to promote vSMC coverage. Regardless, these limitations prevented us from assessing whether restoring arterial smooth muscle would then rescue defects in branching."*

7. This group has focused extensively on the various stromal derivatives although with this manuscript they have lumped all the distinct derivatives into a single Foxd1 positive package. A more in depth discussion of the derivatives and potential interface with Netrin-1 signaling is required.

We thank the reviewer for bringing this up. The reviewer is absolutely right that we and many others have treated the kidney stroma as a single Foxd1^{+} population. This is due to extensive lineage tracing studies demonstrating that most of the renal stroma is derived from the population of stromal progenitors found in the cortex and marked by Foxd1 expression (Kobayashi et al., 2014).

However, we now know that the Foxd1-derived population is not in itself a homogeneous population, but instead is comprised of a number of different cell types, as shown in our recent paper (England et al., 2020). This includes many of the mural cell subtypes we interrogate in this paper, such as the vascular smooth muscle and pericytes. In the current manuscript, we underscore the heterogeneity of the periarterial mural cells by their relative levels of expression of aSMA and NG2, as well as the differential mural cell phenotypes observed in our knockouts (please see **Fig. 5C-E**, as well as discussion on page 8).

Page 8: *"Costaining for aSMA showed that inner layers of SMCs expressed NG2 (**Fig 5C'**, white arrowhead), but outer layers of mural cells expressed only NG2, with little to no aSMA* ***(Fig. 5C'****, green arrowhead). NG2^{+} mural cells were still able to invest arteries in Ntn1^{SPKO} kidneys but appeared markedly reduced* ***(Fig. 5D'****, red arrowheads). … Interestingly, costaining with aSMA (magenta arrowheads) showed incomplete tcolocalization with ectopic smooth muscle at the surface. These data suggest that NG2^{+} cells around arteries in Ntn1^{SPKO} kidneys are distinct from vSMCs. These cells are also mislocalized without Ntn1, but are not completely gone from arteries."*

In addition, whether the cortical Foxd1 progenitor population is multipotent or itself is heterogeneous and has multiple predetermined fates is unknown and hotly debated. We are of the opinion that the progenitor population is heterogeneous, and indeed we present in this manuscript some of the first evidence (that we know of) that there are subsets of progenitors that give rise to specific mural cell types (stromal derivatives). Here, we suggest that Klf4 expression, based on its known role in smooth muscle differentiation and the phenotype of its knockout, marks a specific smooth muscle progenitor within the Foxd1 population. Klf4 is expressed stronger in some, but not all Foxd1^{+} progenitors (please see **Fig S6B**), and expression patterns of Klf4 and Ntn1 are overlapping but distinct at E13.5 within the cortical stroma (please see **Fig. S6C**). Therefore, we focus and shed light on the heterogeneity of both the derivatives AND the progenitors of the renal stroma in this manuscript. We have added significant discussion in response to the reviewer's point, especially on particular interactions of Klf4 with netrin-1, throughout the paper. Please see pages 9, and 13 ("Heterogeneity in stromal progenitors and descendants") for added discussion on the issue. New figure **S6** panels **B-C**"' further underscore this point.

© 2023. Published by The Company of Biologists under the terms of the Creative Commons Attribution License (https://creativecommons.org/licenses/by/4.0/).

Page 9: *"This is further evidenced here by stronger Klf4 staining in a subset of Foxd1+ progenitors* ***(Fig. S6B)****. To validate SP heterogeneity by RNA expression, we performed RNAscope expression analysis on E13.5 kidneys. In agreement with in situ hybridization, both Ntn1 and Klf4 are expressed in the cortical stroma* ***(Fig. S6C)****, with little overlap of Ntn1 and Six2 expression in NPCs, or cytokeratin (CK) protein in the UB* ***(Fig. S6C')****. Comparing Klf4 and Ntn1 expression* ***(Fig. S6C")****, we note that Klf4 is expressed strongly on the outermost rim of SPs* ***(Fig. S6C"****, green arrowhead), whereas Ntn1 is expressed further in* ***(Fig. S6C"****, magenta arrowhead). There is significant overlap of expression (green/magenta arrowhead), but expression patterns remain distinct."*

-and-

Page 13: *"Most intriguingly, we observe different levels of Klf4 protein in Foxd1+ progenitors at E13.5. The Foxd1+ SPs were initially described as a multipotent and homogeneous population. However, cells at the kidney surface have more Klf4 protein. Expression of Klf4 and Ntn1 reinforces the heterogeneous nature of the SPs, as Ntn1 is not expressed in the outermost rim of Klf4+ cells, and conversely Klf4 is not expressed in Ntn1+ progenitors found further from the edge of the kidney. … Klf4 protein remains strong in the outermost cells of the kidney throughout development until E18.5… Further studies with higher resolution expression data and new lineage tracing tools will be required to demonstrate specific lineages from progenitor to descendant, but here we present data suggesting a heterogeneous progenitor population."*

8. To really delve into the perceived role of Netrin-1 in driving differentiation and arteriogenesis an in vitro model showing how the knockout cells interface with endothelium and angiogenesis would be informative.

We appreciate the reviewer's point that it would be useful to be able to use an *in vitro*, reductionist model to study how Ntn1 KO cells might interface differently with endothelium during arteriogenesis. However, modeling the mechanism of action of Ntn1 *in vitro* is difficult. First, there are no cell lines available that represent the Foxd1+ stromal progenitor. Ntn1 is not expressed in any stromal derivatives such as smooth muscle or pericytes, for which lines exist, so it would be difficult to create a relevant knockout and control cell lines. Second, we know that biomechanical cues from hemodynamic flow profoundly impact vascular development and likely also the recruitment of smooth muscle/pericytes. As we have recently shown (Ryan et al., 2021), kidney vasculature *in vitro* fails due to lack of blood flow.

Unfortunately, these experiments would be difficult to design, as we have not yet identified the specific downstream receptor or target cell type which netrin-1 signals to (although we have tried, but none of the receptor knockouts mimic the Ntn1 knockout). While endothelial cells could be the downstream target cell for the Ntn1 signal, via the receptor Unc5b, expression of other receptors in other cell types makes it difficult to ascertain the appropriate co-culture system with Ntn1 KO stromal cells. Both we and the O'Brien lab have extensive expression data for the possible Ntn1 receptors showing that at least one receptor is expressed in almost every cell type in the kidney, making a ligand-receptor prediction difficult. As our current model shows, we imagine that Ntn1 may directly communicate with mural cells within the stroma, or it might act on other cell types and secondarily impact mural cells. We now discuss some additional signaling possibilities and possible target cell types (please see page 15).

Page 14: *"We show that Ntn1 has many roles in the developing kidney, including regulating vascular patterning and mural cell differentiation, as well as epithelial morphogenesis (directly or indirectly). Given the expression of multiple known Ntn1 receptors, such as Unc5c, Neo1, and Unc5b, in various potential target cell types throughout the developing kidney, and Ntn1 restriction to the cortex throughout development, more in depth evaluation of Ntn1 signaling is needed. Moreover, some studies have suggested Ntn1 has signaling functions that are independent of known receptors (Wilson et al., 2006), underscoring the difficulty of identifying responsible receptors in the kidney."*

******* Reviewer 2 Comments for the Author:**

The generation of functional cell-derived tissues is dependent on an understanding of the molecular

© 2023. Published by The Company of Biologists under the terms of the Creative Commons Attribution License (https://creativecommons.org/licenses/by/4.0/).

mechanism controlling organ specific vascular patterning. The ms. submitted by Luo et al investigates the role of the secreted signaling factor, Netrin1, in this process as the kidney develops. A cursory analysis of renal epithelial development indicates that an absence of netrin 1 signaling results in small kidney size despite prolonged nephrogenesis. The primary focus of the study is a more thorough analysis of the renal vascular network in the absence of netrin 1 signaling. This part of the study demonstrates that netrin1 plays a role in vascular patterning at early stages of development, and that Klf4 is downstream of netrin signaling.

However, these results are not so interesting as arterial vascular patterning at later stages of development appears to normalize. Due to the complexity of the mature renal vascular bed, a thorough analysis of the normalized arterial tree in the mature mouse is difficult. That the netrin-1 mice live into adulthood suggests that the netrin-1 null vascular network is sufficient for renal function. Blood flow studies at early and late stages of development would greatly strengthen the hypothesis that the vascular network is defective at early, but not late stages of development. Notably, as stated in the discussion these experiments have been performed but are not included in ms.

In summary, this ms. would be of great interest if it investigated the mechanisms guiding the normalization of arterial patterning defects observed at early stages of development. As it stands now, it is focused solely on a transient phenotype. Moreover, many of the experiments are done in a superficial manner and interpretation of some crucial results flawed.

We appreciate the reviewer's critiques of the presented study. It is true that both we and Honeycutt et al present the Ntn1^{SPKO} phenotype of smaller kidneys and altered epithelial development, however, both our studies have focused on how the vasculature is impacted. In part, it is because we both believe the epithelial phenotypes are likely secondary. Most of our data point to Ntn1 loss impacting the stroma directly, or indirectly via an effect on the vasculature. While the most severe arterial patterning defects (presence of collateral arteries and kidney penetrating arteries), and their downstream effects (perfusion and Cx40 positivity) grossly normalize over developmental time in our analysis, we note that by E18.5 arteries do not fully recover and are still thinner at later time points. In addition, the O'Brien group has focused on the arterial branching in postnatal and adult kidneys, and they show that Ntn1 KO kidneys display defective branching at and beyond the timepoints of our study.

It is important to underscore that our groups communicated about our parallel studies over the last few years, after we learned we were in competition as we were studying the same mutant. Over the course of our communications, we decided to work up complimentary studies, rather than attempt to publish overly similar or overlapping studies. The O'Brien lab focused on the postnatal effects, while we studied the prenatal ones. We think that these complementary analyses have made a strong case that netrin-1 is critical for early branching of the renal artery via its impact on the stroma. We show that loss of Ntn1 results in altered vessel morphology of the early renal artery, resulting in extrarenal and penetrating arteries. To the reviewer's point, we have added data showing altered perfusion of blood vessels only at early stages in Ntn1 mutants (please see **Fig 3C,D, Fig. S3K,L, Fig S7C,D**), and point to the O'Brien lab's manuscript for perfusion data at later stages (Evan's blue is perfused into the arteries in their studies). Along with the O'Brien lab's detailed analysis of post- natal vasculature, we show that loss of Ntn1 continues to affect branching of the arteries within the embryonic kidney (please see new **Fig. S3Q)**, potentially secondary to mural cell coverage (please see new **Fig. S4E**). Thus, while many of the ectopic vessels and connections may regress as the embryo grows, many phenotypes persist. As such, the fact that the Ntn1 knockout mice survive into adulthood does not in our eyes diminish the importance of our study, as we do not know yet know whether they may experience worse renal function upon stress i.e. IRI, or potentially experience diminished life spans. These adult studies would be extremely interesting, but outside the scope of our developmental studies. To address the reviewer's point, however, we have added discussion including progressive restoration of blood flow on page 11.

Page 11: *"We find that the stereotyped pattern of arterial connection of the kidneys to the aorta is lost in Ntn1 mutants. These defects are accompanied by a decrease in perfusion and flow response within early arteries. Ntn1^{SPKO} mutants exhibit excessive branching of*

© 2023. Published by The Company of Biologists under the terms of the Creative Commons Attribution License (https://creativecommons.org/licenses/by/4.0/).

the renal artery between the aorta and kidney, suggesting either an increase in angiogenic activity or a failure of vessels to remodel into a singular renal artery. This abnormal pattern is associated with arteries entering the kidney farther from the hilum, resulting in decreased perfusion and stunted growth of arteries within the kidney. We show this decreased blood flow both with IB4 perfusion into the embryonic vasculature, as well as by flow-dependent Cx40 expression. How Ntn1 affects remodeling of arteries outside the kidney is unknown, but it is possible that Ntn1, secreted by SPs at the earliest stages of nephrogenesis into the angioblast-rich mesenchyme, regulates remodeling of the renal artery as it sprouts from the aorta.

The effects of early mispatterning in mutants is progressively minimized as the kidney grows and relative arterial distances from the hilum become closer to wild type. This is accompanied by increased perfusion and Cx40 expression in all arteries by E15.5, potentially due to increases in blood volume and pressures in the embryo or pruning of ectopic vessels that shunt blood away from the kidney."

In addition, the embryonic phenotype of stromal dysfunction and premature smooth muscle differentiation is extremely interesting to us. We note that smooth muscle coverage continues to be decreased at E15.5 (please see **Fig. S4C-F**), and the O'Brien lab's manuscript shows this is not transient and continues into adult stages. In addition, ectopic smooth muscle cells remain at the kidney surface, despite restored coverage of arteries at E15.5, showing that the effects of Ntn1 loss are not resolved. Most importantly, per suggestions of the other reviewers, we have added data to show that the loss of smooth muscle alone caused by the stromal Klf4 knockout is enough to delay branching of the renal arteries at E15.5, despite apparent recovery of smooth muscle coverage. We have discussed these results in detail, as well as their potential relation to arterial mispatterning, on page 12.

Page 12: *"An important finding from our work and the work of others (**Honeycutt, et al**) is decreased arterial branching within the kidney without Ntn1. It is possible that the aforementioned alterations in early arterial patterning, and resulting decreases in perfusion, have downstream effects on branching within the kidney. However, we propose that arterial branching is instead regulated by vascular smooth muscle coverage. Recruitment of mural cells such as vSMCs impacts vascular integrity and function, as well as vascular patterning (Armulik et al., 2011; Gaengel et al., 2009; Kemp et al., 2022; Orlich et al., 2022; Stratman et al., 2017). We find that arterial smooth muscle coverage is decreased without Ntn1, and Honeycutt, et al find that both smooth muscle coverage and arterial branching are affected well into adulthood, making it unlikely that the transient loss of flow during early nephrogenesis is the root cause. Furthermore, we find that Ntn1 promotes smooth muscle coverage of arteries by suppressing the differentiation of smooth muscle progenitors at the cortex via Klf4. Ablation of Klf4 from the renal stroma results in similarly decreased smooth muscle coverage and arterial branching, but does result in alterations in early renal perfusion. This shows that ramified arterial branching is independent of early artery mispatterning downstream of loss of Ntn1. These findings are in line with previous characterizations of smooth muscle-related stromal mutants and their effects on arterial branching (Hurtado et al 2015)."*

Together, this work identifies a critical period during nephrogenesis, and the ability of the kidney to recover, reflects the resilient nature of the renal stroma. We agree with the reviewer that mechanisms underlying the partial recovery of smooth muscle coverage despite progenitor depletion are incredibly interesting, and again, likely point straight to the resilience of the stroma. Further studies will be needed to elucidate these mechanisms. We have added discussion on potential mechanisms and cell types involved on page 13.

Page 13: *"A final manifestation of the heterogeneous stroma is the nature of the smooth muscle cells seen in later stages of Ntn1^{SPKO} and Klf4^{SPKO} kidneys. Despite continued presence of ectopic smooth muscle at the surface, arteries are covered with some (albeit significantly less) smooth muscle in these mutants. These vSMCs, while likely not derived from the original dedicated smooth muscle progenitors, could still originate from the cortical stroma as other cells may be able to compensate. Alternatively, the NG2^{+} cells*

© 2023. Published by The Company of Biologists under the terms of the Creative Commons Attribution License (https://creativecommons.org/licenses/by/4.0/).

around the arteries in Ntn1^{SPKO} may transdifferentiate into smooth muscle and express aSMA. Another possible source is from outside of the kidney, such as the aortic smooth muscle or the Tbx18^{+} progenitors found in the ureter, which has been described to also give rise to vascular smooth muscle (Airik et al., 2006), but the contributions of these sources in control and mutant kidneys is still poorly understood. In support of one of these options, it appears that penetrating arteries have stronger aSMA staining that decreases as they travel into the kidney, suggesting that they bring in their own smooth muscle that could proliferate and ultimately cover the rest of the arterial tree, effectively rescuing the phenotype. No matter the source, this compensation demonstrates the resiliency of the renal stroma and reflects its importance to kidney development."

Specific comments:

- "Loss of renal netrin-1 impairs recruitment of arterial smooth muscle and premature differentiation at the surface of the kidney" More definitive smooth muscle markers such as <u>myh11 or calponin</u> are required to support the hypotheses that that netrin1 ablation results in ectopic localization and precocious smooth muscle differentiation.

We thank the reviewer for this suggestion. We have carried out new immunofluorescent antibody stains for CNN1 (in whole mount) at E13.5 (<u>please see **Fig. 6C,D** and discussion on pages 8 and 9</u>). Results with CNN1 show that this definitive smooth muscle marker is ectopically expressed by cells at the kidney surface when Ntn1 is absent, similar to what we had seen with aSMA. Unfortunately, we could not get MYH11 to work with our protocols. Of note, Cnn1 and aSMA do not perfectly overlap, suggesting some heterogeneity in the phenotype and potential subtypes of mural cells accumulated at the surface of the kidney. We believe these data support our hypothesis that stromal progenitor cells are precociously differentiating to smooth muscle in the absence of Ntn1.

<u>Pages 8-9:</u> *"We performed WMIF for CNN1, a regulator of smooth muscle contractility and marker of differentiated SMCs, and found that it was upregulated at the kidney surface **(Fig. 6C,D)**. Many ectopic cells in mutant kidneys co-expressed CNN1 and aSMA **(Fig. 6D')**, but the presence of cells only expressing one marker **(Fig. 6D",D"')** suggested a heterogeneous response to the lack of Ntn1."*

- Although Connexin40 expression seems to be decreased in mutants at 13.5, differential expression at later stages of development is not reported.

We thank the reviewer for pointing this out. We have now added Cx40 stains at E15.5 showing that arteries are broadly Cx40^{+} at later stages (<u>please see **Fig S3I,J**</u>). In line with our previous discussion, we think that the decreased Cx40 in intrarenal arteries at E13.5 is a consequence of blood shunting into collateral branches at this earlier stage, delaying blood flow and arteriogenesis. Such anatomical abnormalities are likely to reduce hemodynamic flow temporarily. To address this point, we have now included perfusion experiments (<u>please see **Fig. 3C,D, Fig S3K,L** and **Fig. S7** C,D</u>) which lend support to this idea that blood flow and Cx40 expression are linked (also see our previous work Chong et al., 2011. <u>We have also added discussion on this point on pages 5 and 6</u>.

<u>Page 5:</u> *"As Cx40 is expressed in arteries in response to blood flow (Chong et al., 2011), we injected fluorescently tagged isolectinB4 (IB4) into the embryonic vasculature at E13.5 to assess vessel perfusion. In control kidneys, IB4 remained within renal arteries, but in Ntn1^{SPKO} kidneys, no specific signal was evident within most of the kidney, instead we noted IB4 perfusion of large vessels outside the kidney **(Fig. 3C,D)**."*

-and-

<u>Page 6:</u> *"Next, we analyzed the progression of defects in proximal artery patterning and invasion. Intriguingly, arteries in control and Ntn1^{SPKO} kidneys exhibited similar levels of Cx40 immunostaining and IB4 perfusion by E15.5 **(Fig S3I-L)**."*

- The Klf4 rescue experiments are very superficial. Is kidney size also restored?

We recognize that our initial characterization of the Klf4 rescue was limited to analyzing the

© 2023. Published by The Company of Biologists under the terms of the Creative Commons Attribution License (https://creativecommons.org/licenses/by/4.0/).

ectopic smooth muscle at the surface. Expanding our analysis of the Klf4 rescue was also brought up by Reviewer 1 (please see rev 1 point 6 above).

To address the reviewers' questions, we have added data showing that overexpression of Klf4 in the Ntn1 stromal KO does not in fact restore kidney size, nor arterial branching. Hence the Ntn1-Klf4 axis is limited to smooth muscle cell fate. Regarding arterial branching in the rescues, we note that the nature of the overexpression of Klf4 not only restores Klf4 levels in the stromal progenitors at the surface, but also throughout all Foxd1Cre stromal derivatives, including PDGFRb^{+} mural cells around the artery. This complicates the rescue, as smooth muscle cells would not be able to differentiate at the arterial surface with ectopic Klf4, so we chose to focus only at the kidney surface. We have included this discussion and potential mechanisms on page **Fig. S6 H-K** and discussed it on page 10.

Page 10: *"Klf4 overexpression also did not rescue gross arterial patterning defects* ***(Fig. S6H-J)****, nor did it restore kidney size in the Ntn1^{SPKO} background* ***(Fig. S6K)****, suggesting these effects of Ntn1 loss were independent of Klf4."*

******* Reviewer 3 Advance Summary and Potential Significance to Field:**

Luo et al. investigate the molecular mechanisms that direct vascular patterning of the developing kidney. They focus on the guidance molecule Netrin1, and generated and analyzed mice harboring a stromal- progenitor Netrin1 KO. They do a very thorough job of describing the multiple defects resulting from this deletion, that affect kidney size, epithelial branching and nephrogenesis, as well as angiogenesis and arteriogenesis. They find that multiple renal cell types are affected by loss of stromal Netrin1, including epithelial cells, endothelial cells, pericytes and vascular smooth muscle cells. Bulk RNA sequencing identified KLF4 downregulation in Ntn1 kos, and a stromal progenitor KLF4 knockout phenocopies the Ntn1 ko. Further, conditional KLF4 overexpression rescues smooth muscle cell differentiation in Ntn1 mutants.
The authors propose a novel stromal-derived Ntn1-KLF4 axis that regulates proper nephrogenesis and vascularization of the embryonic kidney. The work is well written and illustrated, and reveals for the first time a vascular phenotype in Ntn1 mutant kidneys. This phenotype is complex, therefore mechanisms of action might be difficult to determine, as exemplified in the accompanying paper of Honeycutt et al. Nevertheless, assembly of organ-specific vasculature remains poorly understood, and this work is important as it reveals novel roles of Netrin1 in renal vasculature development.

Reviewer 3 Comments for the Author:
Before publication can be recommended, the following issues should be addressed.

1- Vascular phenotypes in stromal Ntn1 mutants are shown to be present at E13.5 but are resolved at E18.5 in this manuscript. Honeycutt et al observe persistent defects at postnatal stages, please clarify. Likewise, please provide quantifications for vascular branchpoints and coverage at different time points. Honeycutt Fig.4 shows similar values for vasculature between control and mutant at E15.5, which is at odds with the severe arterial branching defects shown in this manuscript.

We thank the reviewer for these positive comments and these questions. To answer the first point, we would like to clarify the two broad effects we think Ntn1 has on the arteries of the kidney. The *first* is focused on early development, where we see that lack of Ntn1 results in gross patterning defects such as extra-renal and penetrating arteries, which result in decreased blood flow into the kidney (please see **Fig 3A-I, S3A-H**). The data suggests that netrin-1 is required for early arterial patterning, acting on the renal artery as it branches off the aorta, as well as throughout development, via its influence on stroma that surrounds the kidney. We find that the effects of these defects are mitigated over time by the growth of the kidney, leading to restoration of blood flow and downstream targets such as Cx40 by E15.5 (please see **Fig S3I-L**).

However, as the reviewer points out, Honeycutt et al observe persistent decreases in branching and smooth muscle coverage. We think this represents a separate and *second* process mediated by Ntn1's effects on the renal stroma. As requested, however, we have

© 2023. Published by The Company of Biologists under the terms of the Creative Commons Attribution License (https://creativecommons.org/licenses/by/4.0/).

added quantifications to show decreased smooth muscle coverage and branching in the mutant, agreeing with Honeycutt et al's characterization at E15.5 (please see **Fig. S3Q** and **Fig S4E,F**). The original Honeycutt Fig. 4 shows no significant change in branching when looking at the CD31$^+$ vasculature (panel 4B, new panel 5B), but significant decreases in branching and total vasculature when looking at aSMA-covered vasculature (panel 4D, new panel 5D), similar to our aSMA stainings. In particular, our data regarding Sox17$^+$ arteries show that some ectopic arteries in the mutant are not covered by smooth muscle at E15.5. Thus the studies are concordant. It is important to note that our focus on earlier stages allowed us to examine vascular defects likely resolving by E15.5

Both our labs have come to believe that smooth muscle coverage is supportive of branching and persistence of renal vessels. We have worked together to clarify our discussions of our data throughout the text, to make them more congruous and interpretation easier (see section "*Ntn1 promotes ramified arterial branching by regulating smooth muscle differentiation*" on page 12).

2- The mechanism by which Ntn1 regulates Klf4 remains rather elusive and should be improved. Klf4 was shown to regulate Netrin-1 promoter activity in other cell types (doi: 10.1038/ncomms8398). The authors claim that KLF4 is downstream of Ntn1, but they should also check Ntn1 levels in Klf4SPKO and cre- kidneys.

We agree that the mechanisms by which Netrin-1 regulates Klf4 expression not entirely resolved by the present manuscript. Despite adjacent localization of netrin-1 protein around the Klf4+ stromal cells, we do not believe that this is a direct induction. We have tested treatment of HEK293T cells with recombinant Ntn1 and did not see Klf4 induction by a luciferase reporter assay. We opted to not include this negative data due to potential shortcomings in our *in vitro* model system choice.

However, in combination with the lack of specific known Ntn1 receptor expression in Klf4$^+$ stromal cells, we believe that netrin-1 may induce or maintain Klf4 expression via a non-cell autonomous relay mechanism. We have expanded our discussion (please see last paragraph of discussion section, page 15-16) to elaborate on specific possibilities. See also our response to point 6, for more discussion on Klf4 regulation with and without netrin-1.

We also agree that induction of Ntn1 expression by Klf4 would be an interesting and novel feedback mechanism. We have added new data showing that at E13.5, ablation of Klf4 in the Foxd1 stromal progenitors does not result in decreased Ntn1 staining by IF (please see **Fig 7A,B**). This suggests that Klf4 is not required for netrin-1 expression, however, it is possible that Klf4 induction of Ntn1 occurs outside of the timeframe that the Foxd1Cre is able to ablate Klf4.

In addition, we argue that if Klf4 did induce netrin-1 expression directly, it would likely be one of many factors regulating its expression. To support this, we point out that while Klf4 protein is diminished strongly from the stromal fraction by E15.5 in wildtype kidneys, we and others have observed Ntn1 expression by *in situ* hybridization as late as E18.5. This observation does not preclude the possibility that Klf4 may be responsible for early netrin-1 expression, but not later expression. We agree more work needs to be done to clarify the upstream regulation of netrin-1 expression. Discussion on this point has been added on page 14.

Page 14: *"Interestingly, in other systems Klf4 has been shown to promote transcription of Ntn1 (Orgeur et al., 2018). This would represent an intriguing feedback mechanism whereby vSMC progenitors utilize Ntn1 to help control their differentiation state. We observe that Klf4 and Ntn1 are sometimes expressed in the same cells, but the most cortical SPs express only Klf4, and progenitors further from the surface express only Ntn1. In addition, loss of Klf4 from the Foxd1$^+$ SPs alone does not result in loss of Ntn1 protein."*

4- Are KLF4 levels normalized over time in Ntn1SPKO kidneys, along with their phenotype?

© 2023. Published by The Company of Biologists under the terms of the Creative Commons Attribution License (https://creativecommons.org/licenses/by/4.0/).

Similarly, is the phenotype seen in KLF4SPKO transient like the one seen in NTN1SPKO kidneys?

Great questions. Expression of Klf4 is tightly regulated and we think specific to smooth muscle progenitors within the stromal progenitor pool, reflecting its importance as a transcription factor regulating cell fate. We have added data showing Klf4 protein levels in both normal and Ntn1 mutants at E15.5 and E18.5 (please see **Fig. S6D-G**). In control kidneys, Klf4 is no longer strongest in the stroma but rather enriched in the endothelium of the arteries and glomeruli by E15.5, and by E18.5 is primarily found in the endothelial cells with very little stromal expression. In the *Ntn-1*^{SPKO}, we observe a potentially minor decrease in stromal Klf4^{+} nuclei, but were not able to quantify significantly owing to the relative paucity of stromal Klf4.

Of interest, we point out that there is no effect of loss of netrin-1 on arterial or glomerular Klf4, which is expected as the endothelial cells are not derived from the Foxd1^{+} progenitors. Klf4 expression in arteries is shown to be upregulated in response to blood flow, and thus probably unrelated to the netrin-1 dependent regulation in the stromal progenitors. All this data is supported by the fact that despite Klf4 being down in our early (E13.5) RNA sequencing, Klf4 does not appear to be downregulated in the bulk RNA sequencing experiment done by Honeycutt et al. We interpret this to reflect the fact that other undisturbed sources of Klf4 likely drown out any residual difference by E15.5, when their experiment was done. In sum, we believe that netrin-1 only regulates levels of Klf4 in stromal progenitors specifically not its expression in endothelial cells later in kidney development.

Regarding the second question: We want to clarify that the smooth muscle phenotype we see is not transient, as we see persistence of ectopic smooth muscle cells at the periphery, despite partial restoration of smooth muscle coverage later in kidney development. To us, this suggests an alternate source of smooth muscle progenitors that kicks in to compensate for loss of Ntn1 and Klf4, and highlights the potential resilience of the stroma. To answer the reviewer's question, however, smooth muscle coverage does partially recover at E15.5 in the *Klf4*^{SPKO} mutants (please see **Fig. 7E,F**), but is significantly less when analyzing aSMA^{+} arterial length **(**please see **Fig. 7I)** , despite Cx40^{+} arterial length being not significantly different **(**please see **Fig. S7G)**. In addition, we show that these decreases in smooth muscle likely result in decreases in arterial branching, independent of decreases in perfusion that we don't observe in the *Klf4*^{SPKO} mutants (please see **Fig. 7J)**. We describe these findings on page 10 and discuss it in depth in the section "Ntn1 promotes ramified arterial branching by regulating smooth muscle differentiation" in the discussion.

Page 10: *"At E15.5, arteries in Klf4*^{SPKO} *kidneys exhibited decreased smooth muscle coverage, as measured by total length of smooth muscle covered arteries in aSMA WMIF.* ***(Fig. 7E,F,I).*** *To verify that loss of stromal Klf4 did not affect overall arterial development, we measured total arterial length using Cx40 WMIF at E15.5 and found no significant difference* ***(Fig. 7G,H, Fig. S7G).***
… To test whether loss of smooth muscle coverage alone is sufficient to delay branching, we counted primary branches in E15.5 Klf4^{SPKO} *kidneys by Cx40 WMIF. Similar to the Ntn1*^{SPKO}*, there were fewer branches of main arteries without Klf4* ***(Fig. 7J).****"*

5- The authors state that both Ntn1SPKO and KLF4SPKO kidneys exhibited penetrating arteries that looped and connect to intrarenal arteries in some cases, presence of extrarenal arteries and ectopic SMA coverage. It would be useful to quantify these phenotypes in both NTN1SPKO and KFL4SPKO to understand to which extent these genetic deletions phenocopy each other.

We thank the reviewer for this suggestion, which helps to make our story clearer. We have added data showing that the *Ntn1*^{SPKO} and *Klf4*^{SPKO} do indeed exhibit different phenotypes when looking at arterial patterning and have changed the wording in the text to match (please see **Fig. 3A-D, S3C,D and Fig. S7A-D,F** and text revision on page 10). We see less severe patterning defects of the renal artery in the E13.5 *Klf4*^{SPKO} mutant, and as a corollary, we see that arteries in this mutant do not have decreased Cx40. We discuss these findings on page 10.

© 2023. Published by The Company of Biologists under the terms of the Creative Commons Attribution License (https://creativecommons.org/licenses/by/4.0/).

Page 10: *"Notably, loss of stromal Klf4 caused less severe defects in early (proximal) arterial patterning than Ntn1^{SPKO}, as there was no significant difference in Cx40^{+} arterial length within E13.5 Klf4^{SPKO} kidneys **(Fig. S7A,B,F)**, and arteries were also perfusable by IB4 **(Fig. S7C,D)**. These results suggest that Ntn1 affects early gross patterning by Klf4-independent mechanisms."*

However, the lack of arterial phenotypes in the *Klf4^{SPKO}* fits with our model that Klf4 is responsible for only smooth muscle differentiation within the Foxd1^{+} cortical stromal progenitors. We have performed quantifications of intrarenal artery density in both *Ntn1^{SPKO}* and *Klf4^{SPKO}* kidneys at E13.5 (please see **Fig. S3C/Fig. S7E**) and ectopic smooth muscle at E13.5 (please see **Fig. 4C, Fig. S7E**). To aid with interpretation of all our results, we have added a table (please see **Fig. S7I**) summarizing the mouse models used in this study and their phenotypes.

6- The authors claim that the suppression of branching is due to the excess contractile SMC at the developing kidney periphery. Does NTN1;KLF4 OE rescue arterial branching in addition to the rescue of Ntn1SPKO induced aSMA coverage at the kidney periphery? Overall, a more detailed characterization of NTN1;KLF4 OE kidneys is needed to understand the extent of KLF4 involvement in NTN1-induced vascular patterning of the developing kidney.

Reviewer 1 (point 6) also requested more information regarding the Klf4OE system. We have now added data to show that overexpression of Klf4 alone in the stromal compartment does not rescue either kidney size (assayed at E13.5 by measurement of kidney volume from whole mount immunofluorescence imaging) or arterial patterning (at E13.5, assessed by Nrp1 immunostaining, please see **Fig. S6H-K**) (please also see page 10). In addition, we have now adjusted our discussion of the causes of decreased kidney size, as these data, in addition to data showing *Klf4^{SPKO}* kidneys are not different in size, suggest that it is not due to excess contractile smooth muscle as previously suggested (please see **Fig. S7H**).

Page 10: *"However, by contrast to loss of Ntn1 alone, E15.5 Klf4^{SPKO} kidneys were not smaller than controls **(Fig. S7H)**, suggesting that the decrease in kidney size upon loss of Ntn1 is not due to excess contractile smooth muscle or decreased arterial branching, as these defects are found in both Klf4^{SPKO} and Ntn1^{SPKO} kidneys."*

Of note, we were unable to assess the effects of Klf4 rescue on arterial branching, as Klf4 overexpression did not rescue arterial smooth muscle. This is likely because Klf4 was expressed ectopically in all derivatives of the Foxd1Cre, including PDGFRb^{+} mural cells, making them unable to then differentiate and affect arterial branching. Please see the discussion of the limitations of our overexpression model on page 12. As such, we chose to limit our analysis of this model to the ectopic smooth muscle at the surface.

Page 12: *"Notably, Klf4 overexpression does not restore arterial smooth muscle coverage at E13.5. We suspect that the level of overexpression achieved was enough to reduce ectopic differentiation at the surface, but likely too much to then allow proper downregulation of Klf4 at the arteries and differentiation to vSMCs. Alternatively, as the overexpression was performed in a Ntn1^{SPKO} background, other Klf4- independent effects of Ntn1 ablation could result in a lack of signals to promote vSMC coverage. Regardless, these limitations prevented us from assessing whether restoring arterial smooth muscle would then rescue defects in branching."*

7- Supp Fig S3 G,H and S4 C,D are the same, why? please replace one set of images. Fig.3C and S3C also show same image, please replace one, and provide a table of the number of kidneys analyzed in this work.

We thank the reviewer for pointing out these errors. In both cases, the repeat figures represent a different analysis being done on the same stain and set of kidneys. We have replaced the images in **Fig. S4C,D** with new data. We note that Figure **3C** and **S3C** are not actually the same image - **S3C** and **S3D** are max intensity projections of substacks of Figure **3C** and **3D**, which allows us to clearly see the presence of ectopic arterial connections, as well as

© 2023. Published by The Company of Biologists under the terms of the Creative Commons Attribution License (https://creativecommons.org/licenses/by/4.0/).

lack of progression of intrarenal arteries, that are difficult to see in the main figure. **S3C** and **3C** appear more similar than **S3D** and **3D** because most of the arteries in the control are found within the middle third of the kidney, as opposed to in mutants where many ectopic arteries are found near the periphery. We have left these figures as they were but have added tracing and quantifications for internal and extrarenal arteries to emphasize these points **(Fig. S3C,D).** We have adjusted the text on page 5 to make this clear in both the results and the figure captions.

Page 5: *"Max intensity projections of the center third of Nrp1 WMIFs showed significantly shorter intrarenal arterial length and excess extrarenal arteries in Ntn1^{SPKO} kidneys (Fig. S3A-D)."*

We have provided below a table of the number of kidneys analyzed for each quantification, however all these numbers are provided throughout the text of the manuscript and figure legends.

Figure Number	# kidneys analyzed (mutant)	# unique embryos	# litters represented
**2B (Ntn1 WB)**	3	3	2
**2G (E13.5 UB tip)**	6	6	3
**2I (P5 kw/bw ratio)**	8	4	2
**S2A (E15.5 kl/bl ratio)**	14	7	3
**S2F (P5 Six2 count)**	4	4	3
**S2H (P40 Glom count)**	10	5	2
**S3C/D (E13.5 Intra/extra renal count)**	4	4	3
**S3Q (E15.5 artery branching)**	3	3	2
**4C (E13.5 SMA localization)**	5	5	2
**S4E (E15.5 SMA coverage)**	6	6	2
**S4F (E15.5 SMA coverage)**<br><br>**6G (E13.5 Klf4 punctae)**	3<br><br>9	3<br><br>9	2<br><br>5
**6K (E13.5 Klf4 overexpression SMA)**	6	6	3
**S6K (E13.5 Klf4 overexpression size)**	6	6	3
**7I (E15.5 Klf4KO SMA coverage)**	3	3	2
**7J (E15.5 Klf4KO Cx40 branching)**	3	3	2
**S7E (E13.5 Klf4KO surface SMA)**	5	5	2
**S7F (E13.5 Klf4KO intrarenal arteries)**	3	3	2
**S7G (E15.5 total arterial length)**	4	4	2
**S7H (E15.5 kidney/total length)**	4	4	2

Second decision letter

© 2023. Published by The Company of Biologists under the terms of the Creative Commons Attribution License (https://creativecommons.org/licenses/by/4.0/).

MS ID#: DEVELOP/2023/201884

MS TITLE: Stromal netrin-1 coordinates renal arteriogenesis and mural cell differentiation

AUTHORS: Peter M Luo, Xiaowu Gu, Christopher Chaney, Thomas J. Carroll, and Ondine Cleaver
ARTICLE TYPE: Research Article

I am happy to tell you that your manuscript has been accepted for publication in Development, pending our standard ethics checks.

Reviewer 1

Advance summary and potential significance to field

This manuscript by Luo et al interrogates the role of stroll Netrin-1 in renal arteriogenesis and mural cell differentiation. The authors use a combination of molecular and genetic techniques to define the role of this molecule in kidney development. Much of the field of kidney development focuses on the role of the various kidney progenitors with few focusing on the kidney developing vasculature. This field aptly describes normal vascular formation with sophisticated imaging techniques and further elaborates the cellular interactions that take place between the endothelium and smooth muscle that so closely associates.

Comments for the author

The authors have addressed all my concerns.

Reviewer 2

Advance summary and potential significance to field

The authors have addressed my comments and I am pleased to recommend publications.

Comments for the author

The authors have addressed my comments and I am pleased to recommend publications.

© 2023. Published by The Company of Biologists under the terms of the Creative Commons Attribution License (https://creativecommons.org/licenses/by/4.0/).
